# WittyFit—Live Your Work Differently: Study Protocol for a Workplace-Delivered Health Promotion

**DOI:** 10.2196/resprot.6267

**Published:** 2017-04-13

**Authors:** Frédéric Dutheil, Martine Duclos, Geraldine Naughton, Samuel Dewavrin, Thomas Cornet, Pascal Huguet, Jean-Claude Chatard, Bruno Pereira

**Affiliations:** ^1^ Centre Hospitalier Universitaire de Clermont-Ferrand Service Santé Travail Environnement Clermont-Ferrand France; ^2^ Université Clermont Auvergne, Centre National de la Recherche Scientifique, Laboratoire de Psychologie Sociale et Cognitive équipe Stress physiologique et psychosocial Clermont-Ferrand France; ^3^ Australian Catholic University Faculty of Health, School of Exercise Science Melbourne, Victoria Australia; ^4^ Centre Hospitalier Universitaire de Clermont-Ferrand Service de Médecine du Sport Clermont-Ferrand France; ^5^ Université Clermont Auvergne, Institut National de la Recherche Agronomique, Unité de Nutrition Humaine, Centre de Recherche en Nutrition Humaine Auvergne Clermont-Ferrand France; ^6^ WittyFit Paris France; ^7^ Centre Hospitalier Universitaire de Saint-Etienne, Physiologie de l’Exercice, Laboratoire Interuniversitaire de Biologie de la Motricité Equipe d’Accueil 7424, Université de Lyon, Université Jean Monnet Saint-Etienne France; ^8^ Centre Hospitalier Universitaire de Clermont-Ferrand, Direction de la Recherche Clinique et de l’Innovation Clermont-Ferrand France

**Keywords:** health, work, lifestyle, behavior, management, stress, physical activity, nutrition, sleep, musculoskeletal disorders, depression, anxiety, absenteeism, organization, morbidity, mortality, public health, mhealth, mobile app

## Abstract

**Background:**

Morbidity before retirement has a huge cost, burdening both public health and workplace finances. Multiple factors increase morbidity such as stress at work, sedentary behavior or low physical activity, and poor nutrition practices. Nowadays, the digital world offers infinite opportunities to interact with workers. The WittyFit software was designed to understand holistic issues of workers by promoting individualized behavior changes at the workplace.

**Objective:**

The shorter term feasibility objective is to demonstrate that effective use of WittyFit will increase well-being and improve health-related behaviors. The mid-term objective is to demonstrate that WittyFit improves economic data of the companies such as productivity and benefits. The ultimate objective is to increase life expectancy of workers.

**Methods:**

This is an exploratory interventional cohort study in an ecological situation. Three groups of participants will be purposefully sampled: employees, middle managers, and executive managers. Four levels of engagement are planned for employees: commencing with baseline health profiling from validated questionnaires; individualized feedback based on evidence-based medicine; support for behavioral change; and formal evaluation of changes in knowledge, practices, and health outcomes over time. Middle managers will also receive anonymous feedback on problems encountered by employees, and executive top managers will have indicators by division, location, department, age, seniority, gender and occupational position. Managers will be able to introduce specific initiatives in the workplace. WittyFit is based on two databases: behavioral data (WittyFit) and medical data (WittyFit Research). Statistical analyses will incorporate morbidity and well-being data. When a worker leaves a workplace, the company documents one of three major explanations: retirement, relocation to another company, or premature death. Therefore, WittyFit will have the ability to include mortality as an outcome. WittyFit will evolve with the waves of connected objects further increasing its data accuracy. Ethical approval was obtained from the ethics committee of the University Hospital of Clermont-Ferrand, France.

**Results:**

WittyFit recruitment and enrollment started in January 2016. First publications are expected to be available at the beginning of 2017.

**Conclusions:**

The name WittyFit came from Witty and Fitness. The concept of WittyFit reflects the concept of health from the World Health Organization: being spiritually and physically healthy. WittyFit is a health-monitoring, health-promoting tool that may improve the health of workers and health of companies. WittyFit will evolve with the waves of connected objects further increasing its data accuracy with objective measures. WittyFit may constitute a powerful epidemiological database. Finally, the WittyFit concept may extend healthy living into the general population.

**Trial Registration:**

Clinicaltrials.gov: NCT02596737; https://www.clinicaltrials.gov/ct2/show/NCT02596737 (Archived by WebCite at http://www.webcitation.org/6pM5toQ7Y)

## Introduction

We spend one-third of our lives working. The age of retirement is regularly pushed back [1]. The main challenge in the near future will be to help workers maintain adequate health to do their work until retirement [1]. Morbidity before retirement carries a substantial public health and workplace burden. Therefore, there is a need to promote health globally, and targeting the workplace appears inherently appropriate. Current advances in technology offer infinite possibilities to interact with individuals universally. Software with the capacity to understand an individual within the context of the immediate environment seems to address this challenge. Workplace managers may benefit from advancing their understanding of the perceived well-being of the employees within their company.

The definition of health generated in 1948 by the World Health Organization remains fit for current purposes: “not merely the absence of disease or infirmity but a state of complete physical, mental and social well-being” [2]. Particularly, the role of well-being at work plays a major role in social well-being [3], and research remains limited by few large-scale investigations into physical and mental well-being in this setting. Therefore, we built a software tool based on three major health-related categories derived from the World Health Organization definition of health: physical, mental, and work-related well-being.

Taken separately, there is strong evidence between those factors and health-related outcomes but data are not typically gathered and synthesized in workplace settings. Within physical well-being, we investigate nutrition, physical activity, sleep, and musculoskeletal function. Current evidence strongly supports the benefits of a healthy diet. Multiple aspects of food intake and health have been investigated including the influence of eating breakfast [4] and the effect of excessive dietary sugars [5] and coffee [6] on heart health. The American College of Sports Medicine recommends a minimum of 150 minutes of moderate or 75 minutes of vigorous intensity of physical activity per week to achieve and maintain global health for adults and at least 30 minutes of moderate intensity physical activity per day [7]. Even a smaller amount [8] and even only standing [9,10] without substantially further increasing physical activity have shown benefits on life expectancy. Dose relationships have been identified between physical activity, life expectancy, and health benefits, even when sedentary individuals start to train more intensively after 50 years of age [11]. Therefore, any form of physical activity is better than no activity [7-9]. Similarly, shortened and prolonged sleep durations were associated with increased risk of mortality [12,13]. Also, for musculoskeletal function, the role of pain and pain-inducing musculoskeletal disorders were linked to relationships between perceived health and mortality [14]. By mental well-being, we investigate stress and mood. Stress is now considered a stand-alone risk factor for mortality [15]. Anxiety and depression are common and disabling conditions [16,17]. We investigate work-related well-being with validated and recognized models exploring work organization, job strain, latitude/decision-making, social support, and recognition [18-23].

There is also evidence demonstrating the relationships between physical, mental, and work-related well-being. This is particularly interesting for variables related to work. We will deliberately cite only the most common associations. High job strain and effort-reward imbalance seem to increase the risk of cardiovascular mortality [24]. Some working conditions, such as shift work, have been associated with abnormal eating behavior such as rescheduling of meals and eating different types of food (processed, sweets) [25], promoting an increased risk of developing obesity and metabolic disorders [26]. In addition, stress alone can facilitate unhealthy eating behaviors and the development of obesity [27]. Physical activity is a well-established coping mechanism for stress [28] and mental well-being [29]. Along with reduced workplace absenteeism [29,30], a strong negative association can exist between physical activity and mood states [31]. Working conditions are a strong determinant of morbidity [1,18,19,21,22,32-41]. For example, limited social support at work has been linked with cardiovascular events [42] and depression [43]. Deleterious, contagious effects of poor psychological working conditions are known to be associated with emotional exhaustion and depersonalization [44]. Changes in organization and subsequent conflict of loyalties resulting from work changes can lead to suicide [45]. Ultimately, stress at work is also a complex interplay with sleep [46], diet [47], and physical activity [48,49].

Because physical, mental, and work-related well-being are interconnected, a multifaceted and global understanding of individual perceptions may lead to better and more efficient preventive results. Despite the strong evidence of the benefits of stress management, diet counseling, and exercise training in patients, intervention studies on broad and representative samples of employees remain scarce. Preventive programs specific to work organizations can be implemented at the worksite [50-55]. Whereas other interventional software programs focus on specific problems such as musculoskeletal disorders [56], WittyFit aims to promote health with a holistic understanding of worker health, dynamically aligned to updated scientific knowledge (evidence-based medicine). The development of the health-promoting WittyFit software will be the first interventional study with an epidemiological cohort design that is inclusive of specific details on both leisure time and working conditions, providing a global understanding of individuals. WittyFit software will support recommendations to promote more personalized health prevention initiatives focusing on physical, mental, and work-related well-being. The shorter term hypothesis is that effective use of the WittyFit software will increase well-being and improve health-related behaviors. The ultimate hypothesis of the development of the WittyFit software is that it will increase life expectancy.

Therefore, the overall aim was to build an epidemiological database generated using Wittyfit and combining major lifestyle parameters. We aim to use these data to generate initiatives for decreasing premature mortality and morbidity in conjunction with improved well-being. WittyFit will function to generate a powerful database for strengthening the evidence and advancing knowledge on the relationships between work, behavior, and health derived from a large amount of epidemiological data.

## Methods

### Ethics

This exploratory interventional cohort study in an ecological situation received approval from the ethics committee of the University Hospital of Clermont-Ferrand, France, and has been registered at ClinicalTrials.gov [NCT02596737]. See Multimedia Appendix 1 for the original protocol.

### Participants

All workers agreeing to participate in the WittyFit study will be included from any companies willing to permit their employees to be invited to participate. The WittyFit concept follows an epidemiological design without sample size limitation. Like the Nutrinet-Santé study [57-63], the WittyFit study is never expected to end. Recruitment will commence in January 2016, and data collection will be ongoing due to the prolonged longevity of the project. Duration of participation per individual is unlimited. A staggered strategy will be to target a sample size of 200 workers from Voyages-SNCF.com within the first 6 months in order to improve and evolve the WittyFit software. WittyFit will then extend to other companies in the following year.

### Outcomes

#### Behavioral Outcomes: Three Major Health-Related Categories (WittyFit)

Workers will answer validated questionnaires within three major health-related behavioral categories: physical, mental, and work-related well-being (Textbox 1). Each main category is divided in subcategories:

- Physical well-being investigates nutrition, physical activity, sleep, and musculoskeletal function. Nutrition will be assessed through 24-hour recalls of food intake. Physical activity combines questions about time spent in activity at work and leisure, as well as estimates of sedentary behavior time. This category also includes a questionnaire on sleep quality and quantity [46]. Musculoskeletal function is based on an adapted version of the Nordic Musculoskeletal Questionnaire [64].
- Mental well-being explores stress and mood. We will use the validated Hospital and Anxiety Depression Scale [65] and the visual analog scale of stress [66,67]. Questions on smoking and addiction will be added later.
- Work-related health questions explore job strain, latitude/decision-making, work organization and tasks, social support, and recognition. We will use the validated Job Demand-Control-Support model of Karasek [18-20] and the effort reward model of Sieigrist [21-23] with the addition of some specific questions such as visual analog scales (demand, control, etc) that we want to further validate.

Participants complete questionnaires at times convenient to them; however, participants will be prompted to complete a general visual analog scale from the main categories and major subcategories every 15 days and the more detailed questionnaires every 6 months. When a significant difference appears on the latest visual analog scale, the user is asked to complete specific questionnaires linked with the problem detected by the visual analog scale. Gaming and trophies are incentive strategies for workers to fulfill questionnaires.

#### Economic Outcomes and Outcomes Provided by Employers (WittyFit)

Economic data will be provided by volunteering employers from participating companies: professional roles (unskilled, skilled, mid-level workers, and senior executives), occupational sector, type of contract (full-time, part-time), absenteeism, turnover, and sales revenue and benefits. These data will be continuously updated and monitored. When a participant disappears from the database, the company will provide the relevant reason: retirement, relocation to another company, or death (premature mortality) (Textbox 1).

#### Medical Outcomes (WittyFit Research)

WittyFit is based on two databases: WittyFit, which deals with behavioral data, and WittyFit Research, which deals with medical data. To guarantee the highest level of security, the two databases are separate and do not interact; physicians from the University Hospital of Clermont-Ferrand will be the only researchers with access to medical data. Behavioral data (WittyFit) are those within the three major health-related categories: physical, mental, and work-related well-being. Medical data (WittyFit Research) are medical history and chronic diseases classified using the International Classification of Diseases, medications classified using the Anatomical Therapeutic Chemical classification, any clinical data known by participants such as heart rate or blood pressure, and the most common biological data such as blood glucose levels, hemoglobin A_1c_, or blood cholesterol levels (Textbox 1).

**Parameters measured.**

Data retrieved from self-reported questionnaires:

- WittyFit—behavioral data:
  - Physical health:
    - Nutrition
    - Physical activity
    - Sleep
    - Musculoskeletal function
  - Mental health:
    - Stress
    - Mood
    - Addiction (to be added)
  - Work-related health:
    - Job strain
    - Latitude/decision-making
    - Work orgainzation and tasks
    - Social support
    - Recognition
- Wittyfit Research—medical data:
  - Medical history and chronic diseases classified using the International Classification of Diseases
  - Medications classified using the Anatomical Therapeutic Chemical classification
  - Clinical data known by participants, such as:
    - Heart rate
    - Blood pressure
  - Common biological data known by participants, such as:
    - Blood glucose level
    - Hemoglobin A_1c_
    - Blood cholesterol level

Data retrieved from companies—economic data:

- Continuously monitored:
  - Occupation, type of contract
  - Benefits
  - Sales revenue
  - Absenteeism
  - Turnover
  - Productivity
- When a worker (identified by human resource–generated number) disappears:
  - Retirement
  - Relocation to another company
  - Death (premature mortality)

### Objectives

The shorter term objectives will be to demonstrate that WittyFit improves parameters of well-being and morbidity based on three major health-related behavioral categories. The mid-term objectives will be to demonstrate that WittyFit improves economic data of the companies such as productivity and benefits. The primary long-term objective will be to demonstrate that WittyFit will contribute to decrease premature mortality.

### Confidentiality

All medical data (medical history, treatments, etc) will be only accessible from a carefully restricted WittyFit researcher access platform. All data are anonymous, and the name of the employee is never entered into the database. The database is implemented from a human resource–generated number, which is then automatically converted into another number in the WittyFit database. Data provided by employers (such as professional roles, occupational sector, type of contract, and absenteeism) are automatically associated with the human resource–generated number.

### Intervention

The WittyFit program offers tiered access to employee data for those with middle- and high-level management status.

#### Holistic Understanding of Workers

**Figure 1:**
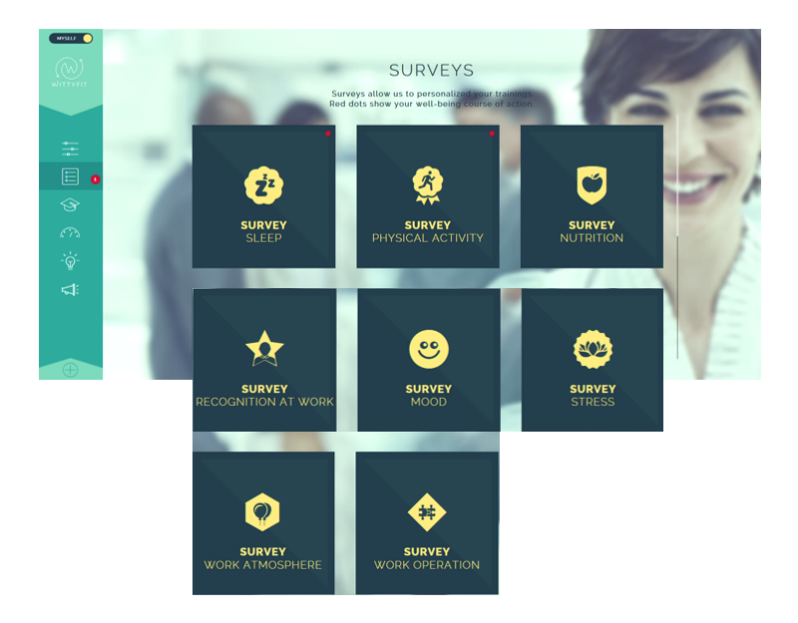
Screen capture of WittyFit: surveys foster a global understanding of workers.

**Figure 2:**
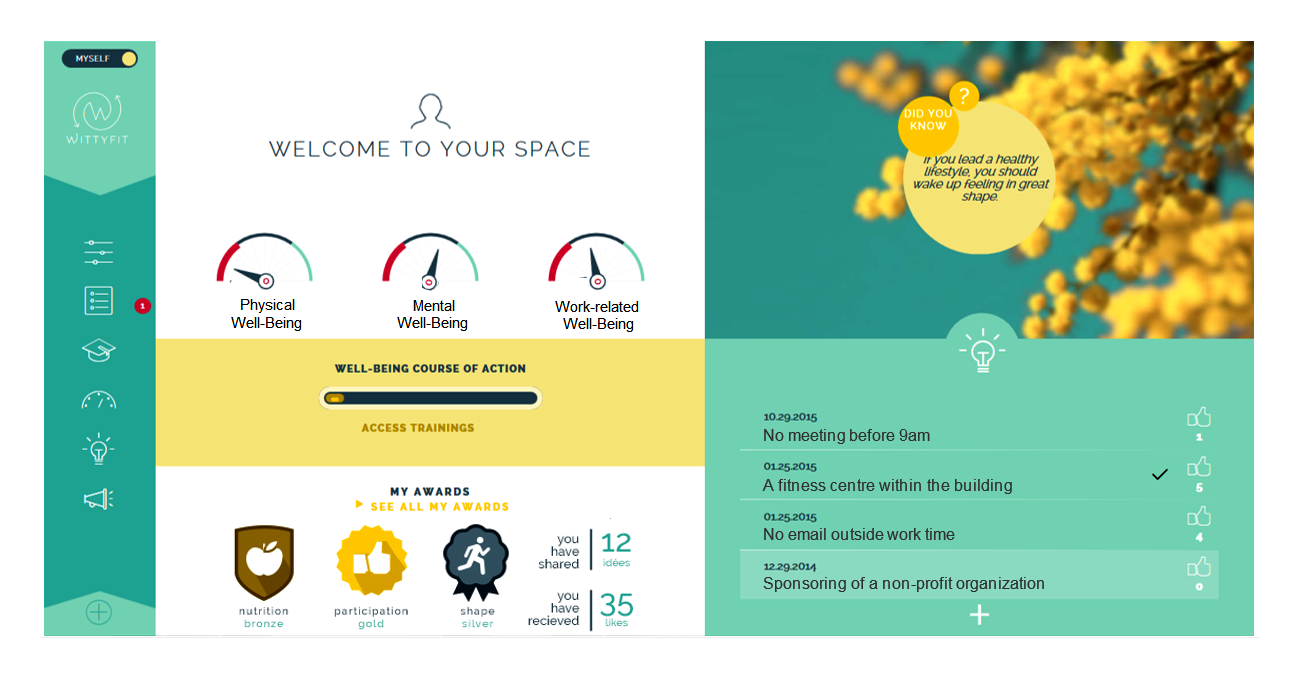
Screen capture of WittyFit: the homepage synthesizing the 3 major health-related categories in a personal dashboard with a menu structure on the left for access to visual analog scales, questionnaires, e-learning sessions, statistics, digital idea box, and polls.

In contrast to other interventional software focusing on a specific aim (such as relaxation or identification of musculoskeletal disorders), WittyFit aims to promote health with a holistic understanding of workers (see Figure 1) based on continuous and updated scientific knowledge (evidence-based medicine). Feedback and counseling are provided on targets (relevant goals) screened from questionnaires (see Figure 2) using an e-learning platform or a “Did you know?” information approach based on personalized analyses of responses to questionnaires.

**Figure 3:**
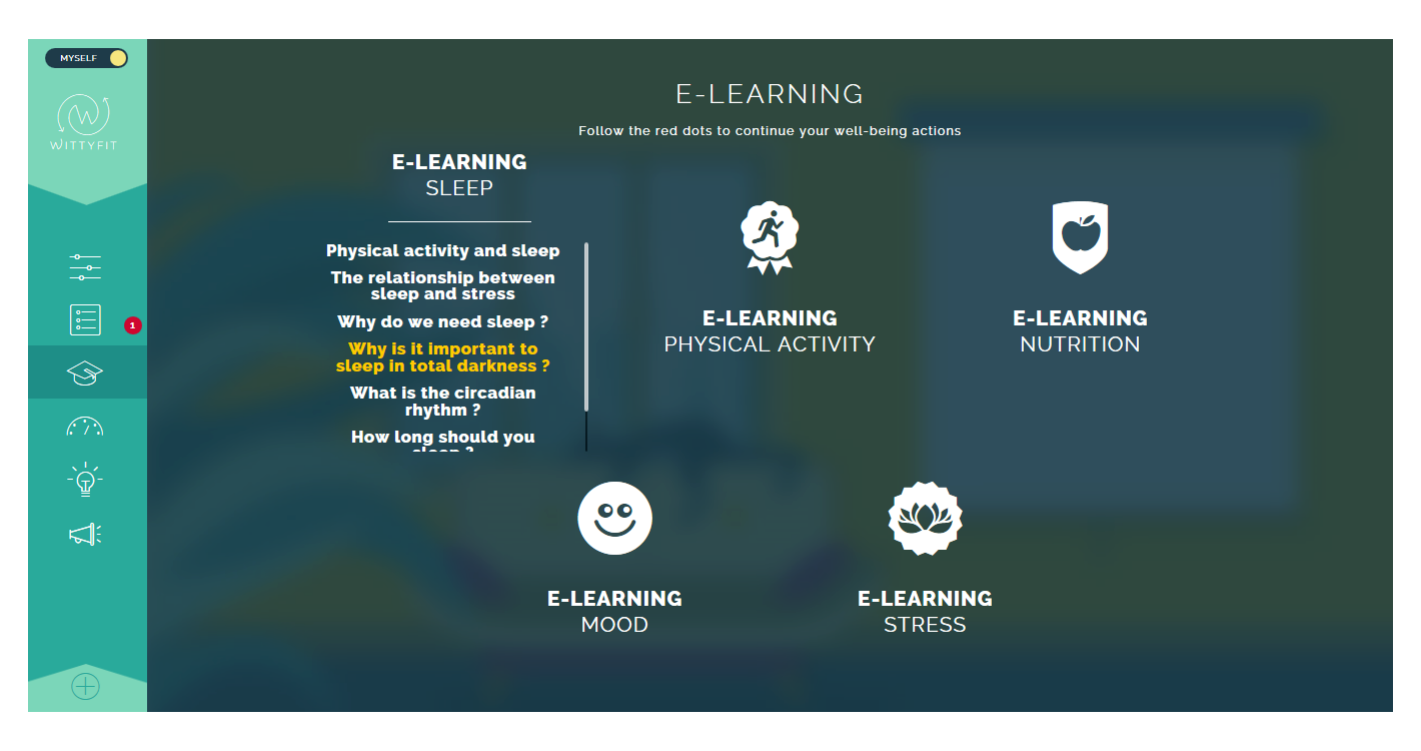
Screen capture of WittyFit: examples of e-learning sessions.

**Figure 4:**
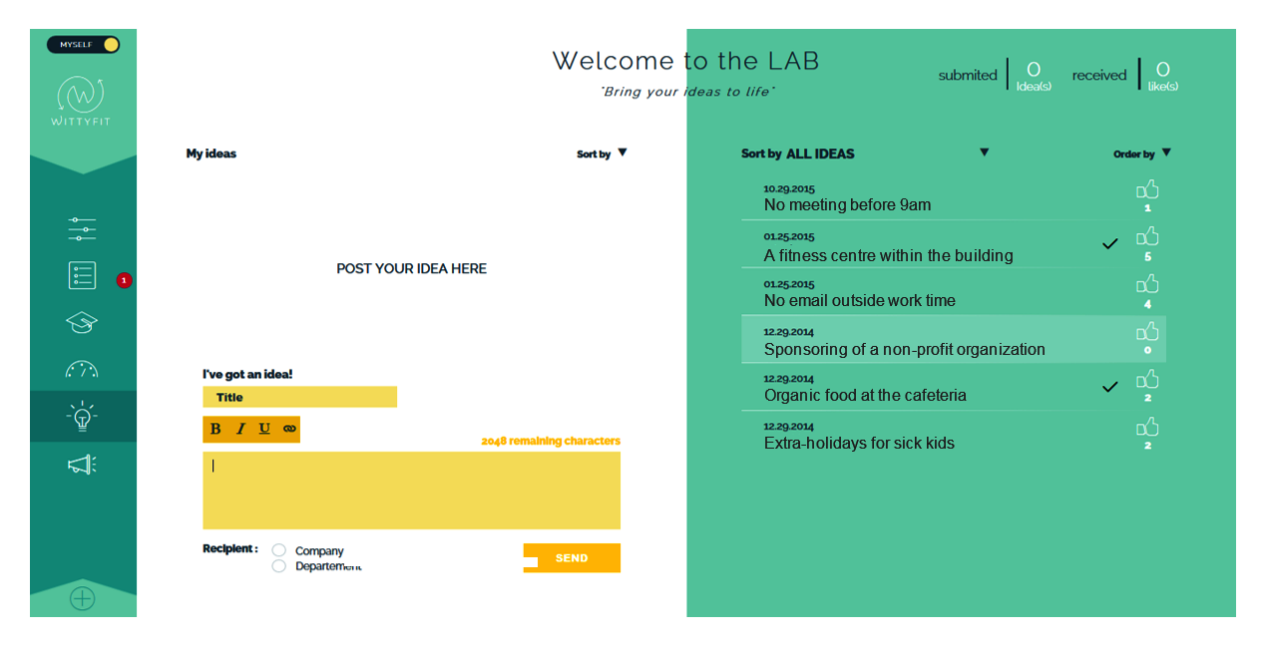
Screen capture of WittyFit: the Digital Idea Box.

Each e-learning module (see Figure 3) is based on a 4-step approach used in pedagogy [68-70]: (1) answer a quiz (pretest), (2) understand the issue, (3) act on the issue, and (4) answer a quiz. The quiz is designed both for learning purposes and to evaluate workers [71]. The number of questions on the quiz is based on learning techniques [72]. The planned process involves the employees accessing their own personal data on a panel of indicators displaying their status, progress, and success. Employees may also contribute new ideas in the “digital idea box” and “likes” about ideas of others (see Figure 4). Employees may participate in company-specific surveys about healthful and feasible changes in the workplace. When some changes for employees are available to consider, a red dot will appear on the access button of the relevant topic.

#### Manager Feedback

**Figure 5:**
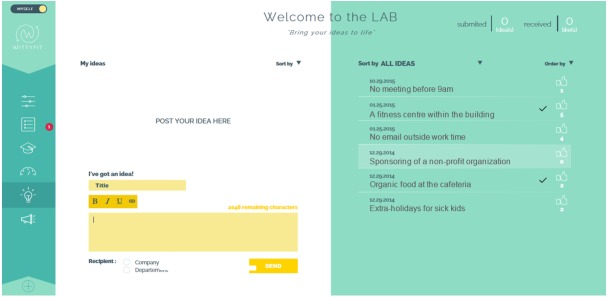
Screen capture of WittyFit access for top management, including anonymous mean level of stress by location, department, age, gender, occupation, etc.

The second feature of WittyFit is that managers will receive anonymous feedback on the general state of health and problems encountered by their employees (see Figure 5) for whole or sectional divisions of the workplace if the sample size is sufficiently high (ratios of 1/10). Middle managers will be able to target specific actions such as promoting physical activity at work or workplace initiatives assisting employees to quit smoking. They will have access to the Best Practice database. Middle managers will also have access to the Forum Manager anonymous community to share their experience. They will have a Go/No Go functionality regarding the proposals in the digital idea box. If they are not in position to decide, the middle managers can propose an idea for the top management. Executive-level managers will have similar access as the middle managers, including indicators by division, location, department, age, seniority, gender, and occupational position. In addition, executive managers will have summaries of the turnover and absenteeism within the context of the visual analog scale data profiles. They can put in motion relevant ideas from the digital idea box and use the “What’s new?” editing tool to predict future changes to the work force in light of the nature of engagement of employees with the WittyFit software.

### Epidemiological Database

WittyFit has the capacity and innovation to strengthen evidence and build new knowledge on the relationships between lifestyle and health based on an increasing volume of epidemiological data.

### Connected Objects

Eventually, WittyFit will further evolve with the waves of connected objective measures of health and lifestyle, further increasing its data relevance, with the use of devices such as pedometers, heart rate monitors, accelerometers, and thermometers. Only volunteering workers will connect these objects to WittyFit. We will soon propose devices able to analyze these objective measures. For example, we have the expertise for objective measure of stress from heart rate variability or skin conductance [73-75].

### Multidisciplinary Project

WittyFit is building a spectrum of health-related data: work (shiftwork, sedentary workplace demands, specific and perhaps high-risk occupations), psychology and physiology, statistics modeling, public health, and medical health.

### Friendly Use

**Figure 6:**
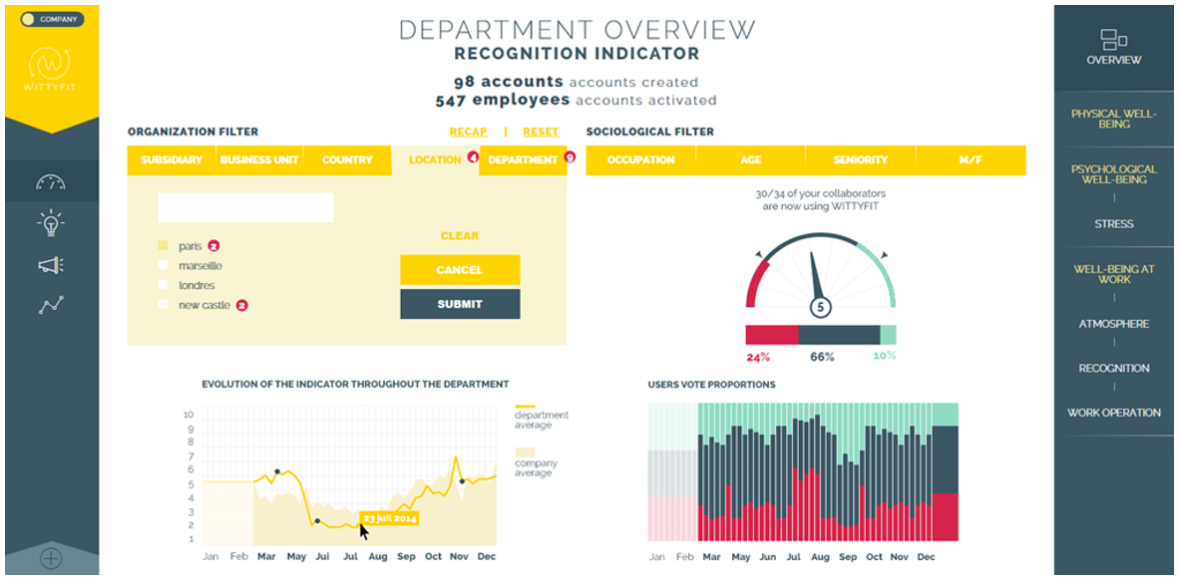
Screen captures of WittyFit mobile app (French version). Left screen: homepage; middle screen: body shape to self-report musculoskeletal disorders; right screen: gaming and trophies are incentive strategies for workers to complete questionnaires.

Ease of use of Wittyfit is our goal. When an action is needed, the user can follow a link from the homepage so there is no waste of time. Users can access previous answers but can only modify specific items. Questionnaires never exceed 12 to 14 questions. Users can stop answering questionnaire and resume later where they left off. At first log-in, completing all questionnaires takes approximately 30 minutes; to reduce the burden, we ask new users to complete all questionnaires over 2 to 3 weeks. Users can refer to an interactive guide that gives them advice on what’s next. WittyFit is available on any device connected to the Internet including computers, tablets, and smartphones; therefore, users can have access to WittyFit from where they want, including outside of their workplace. The gaming process is designed to increase the use of WittyFit by awarding points and badges for participation. The company is free to decide how to reward the best users with innovative strategies such as donations to nongovernmental organizations (see Figure 6).

### Statistical Considerations

#### Sample Size Calculations

Using only Internet or computer interventions for behavior change outcomes improving eating disorders and nutrition [76], weight and cardiorespiratory fitness [77], or mood [78] and stress related to the workplace and performance and productivity [79], the sample size estimation was performed according to Cohen’s recommendations with the following defined effect size (ES) bounds: small (ES 0.2), medium (ES 0.5), and large (ES 0.8, “grossly perceptible and therefore large”) [80]. Considering an effect size of 0.33, a 2-tailed type I error fixed at a=0.001 to take into account multiple comparisons, and a moderate loss to follow-up rate, N=1000 participants will allow us to highlight (1) such ES for a statistical power greater than 95% for paired comparisons (n=225 with a correlation coefficient of .5) and (2) such ES for a statistical power greater than 80% for intergroup comparisons (n=311 by group).

**Figure 7:**
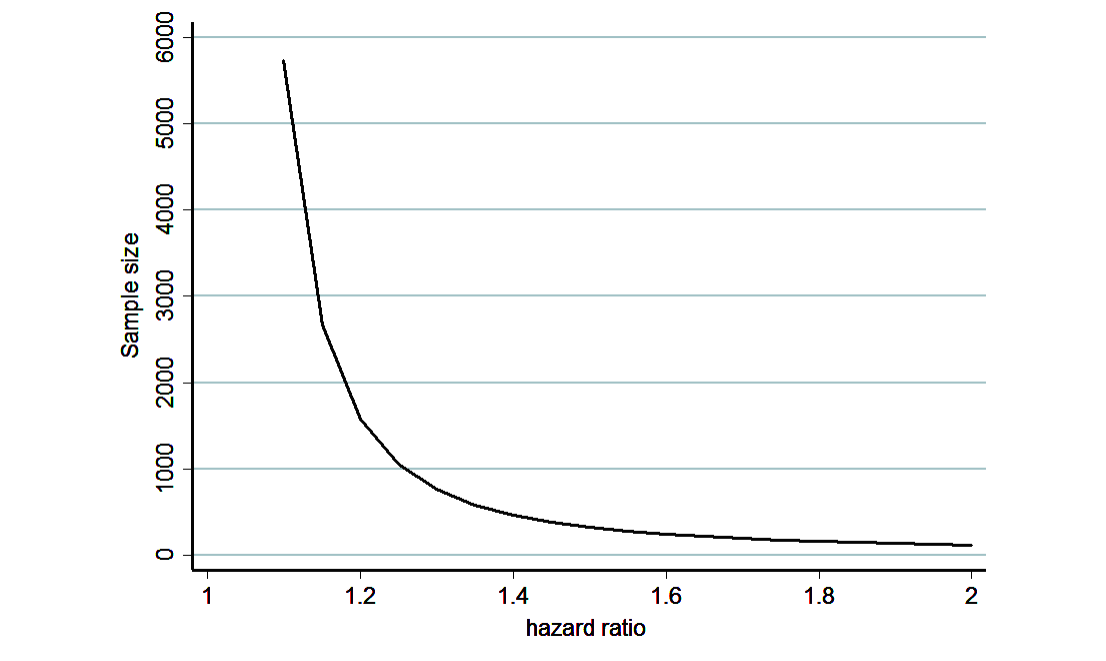
Sample size estimation for life expectancy based on simulations about hazard ratios for censored data as mortality (2-sided type I error of 5% and statistical power 95%).

For life expectancy, 100,000 workers followed over 5 years would provide a sufficient and relevant sample size to achieve the long-term objectives measured by this study. Purposive sampling will be conducted to best draw comparisons with previous research [81] studying leisure time spent sitting in relation to total mortality in a prospective cohort of US adults [9] and to further elucidate job strain among blue-collar and white-collar employees. Job strain has previously been associated with total mortality in a 28-year cohort study [82]. To address the objective of reducing premature mortality, statistical power estimation was based on a hazard ratio used for censored data (hazard ratio of 1.25 and 2) and in accordance with previously described data. This hazard ratio estimate was proposed to support a type I error of 5% (2-sided) with statistical power at 95% (see Figure 7). However, the study will be unlimited within the long-term aspects of its epidemiological design.

#### Overall Statistical Analyses

Statistical analysis will be performed using Stata version 13 software (StataCorp LLC). All statistical tests will be 2-sided, and *P*<.05 will be considered significant. Quantitative variables will be described in terms of numbers, average, median, standard deviation, and range.

#### Analyses of Shorter Term Objectives

To study WittyFit’s effect on morbidity and well-being, analyses will include comparisons with historical cohorts and literature and within the paired data from individual participants as they generate their own basis for comparison. To study longitudinal evolution of parameters associated to morbidity and well-being, models for repeated measures will be performed using a random-effects model (linear or generalized linear) along with fixed effects for descriptive data (eg, gender, age, body mass index, type of contract [full-time, part-time], professional roles [unskilled, skilled, mid-level workers, and senior executives] and occupational sector [commercial activities and service, health and social services, manufacturing/blue collar industries, finance officers]) taking into account within and between participant variability and company variability. All effect sizes will be presented with 95% confidence intervals.

When appropriate, comparisons between subgroups described above will be performed. Typical statistical tests will be applied: chi-square or Fisher exact tests for comparisons between groups from categorical parameters and analysis of variance or Kruskal-Wallis test for quantitative variables. The Shapiro-Wilk test will be used to assess normality, and the Fisher-Snedecor test to assess homoscedasticity. For censored data, the time-to-event curves will be estimated with the use of the Kaplan-Meier method. Comparisons will be performed by log-rank test. Then, Cox proportional-hazards regression models will be considered to study the prognostic factors in a multivariate situation by backward and forward stepwise modeling on the factors considered significant in univariate analysis and in accordance with parameters of clinical relevance [83,84]. The proportional-hazard hypothesis will be verified using Schoenfeld’s test and plotting residuals. The interactions between possible predictive factors will also be tested. Results will be expressed as hazard ratios and 95% confidence intervals. Considering repeated correlated censored data, marginal models may be preferred.

#### Analyses of Longer Term Objectives

Decreased premature mortality is the long-term goal of the development of the WittyFit software. Considering this censored data, estimation will be performed as described previously using the Kaplan-Meier approach. Comparison with historical cohorts and previous research will be included in the estimates of 95% confidence intervals. WittyFit’s effect will be also studied using log-ranked comparisons and a Cox proportional hazards model. Multivariate models will be used according to univariate results and clinical and epidemiological relevance.

#### Method Taking Into Account Missing, Unused, or Invalid Data

A sensitivity analysis of missing data will be applied to assess the level of attrition and to characterize the statistical nature (missing at random, missing completely at random, not missing at random) that will dictate the most appropriate method of imputation [85-87].

#### Quality Control Measures to Reduce or Avoid Bias

The data of employees will be de-identified to assessors when processing data. A large sample size will be employed to justify the validity of the statistical treatment of the data. Generalizability will be ensured via sufficient numbers in each occupational sector to represent targeted sectors.

## Results

WittyFit recruitment and enrollment started in January 2016. First publications are expected to be available in 2017.

WittyFit is a multidisciplinary project. It involves leading specialists in relevant fields including epidemiologists and statisticians, occupational physicians, physiologists, psychologists, psychiatrists, and economists. Implementation and conduct of the study will be monitored by the project management committee (authors) who have extensive experience in research and conducting clinical trials in occupational health as well as statistics and engineering. Outcomes will be disseminated via open-access peer-reviewed publications, conferences, clinical networks, public lectures, and our websites. Community reports on the outcomes will also be made available to participants.

From a workplace and individual perspective, identifying stressful situations at an early stage may avoid socially problematic behavior from occurring and may permit early comprehensive and targeted behavioral intervention. We may identify unpredicted stressful events and recommend appropriate therapies. For the participants, our intervention may permit self-evaluation, giving them the chance to better anticipate and adapt to stressful situations on their own. If this protocol includes heart rate monitoring, it may also incidentally detect cardiac disorders. Such an event will be communicated to the participant with suggestions of supportive strategies. Any abnormality discovered will not be covered by insurance associated with the development of WittyFit but will be supported by typical sources of health insurance.

## Discussion

### Summary

The main aim of WittyFit is to provide early comprehensive and targeted behavioral interventions to improve well-being, morbidity, and ultimately life expectancy. We aim to build an epidemiological database combining major lifestyle parameters including those at work.

Morbidity before retirement has a huge cost, burdening both public health and workplace finances. Therefore, there is an urgent need for a tool such as WittyFit. WittyFit is the synthesis of several novelties. Because all parameters (physical, mental, and work-related well-being) are linked, providing a multifaceted understanding of individuals is needed. Whereas other interventional software focuses on a specific aim (relaxation, identification of musculoskeletal disorders and stress) [56], WittyFit aims to promote health with a holistic understanding of worker health, dynamically aligned to updated scientific knowledge (evidence-based medicine). Feedback is provided on targets screened from questionnaires through e-learning and personalized motivating messages.

The second novelty of WittyFit is that managers will have feedback on the general state of health and problems encountered by their employees (de-identified data), in general or by department if the sample size is sufficiently high. Managers will be able to target specific actions such as promoting physical activity at work or helping employees to quit smoking.

Third, WittyFit is a preventative tool to be used in collaboration with routine occupational medicine. It has the potential for early detection of individuals at high risk of health compromise and the functionality to direct employees to confidential health support services. This can occur by separating data into two platforms: WittyFit, which deals with behavioral data, and WittyFit Research, which deals with medical data.

Fourth, WittyFit will also evolve with more efficient questionnaires. The epidemiological design and the amount of data will permit further sensitivity analysis on questionnaires to select the most discriminant questions and disregard less useful questions. As data collection has already begun, a putative change may appear as a limitation. However, we should be able to compare and follow individuals who have responded to the same baseline questionnaires. WittyFit will have to evolve with connected objects and up-to-date science. For example, calendars of employees may be synchronized with continuous monitoring of stress provided by wrist tools measuring heart rate variability or skin conductance [73-75]. Simplification of questionnaires would therefore be useful and allow the introduction of other measurements such as job satisfaction [88] or work engagement [89]. Such parameters may be considered important aspects of physical, mental, and work-related well-being, as well as workplace productivity [90].

### Potential Limitations

The considerable amount of data may delay comprehensive analysis. However, the first publications will be on easier data processing, validation of questionnaires, and relationships between them. Health interventions designed around health behavior theory seemed most effective in changing behavior [91]. Content analyses of health software indicate that the software generally contains low levels of health behavior theory or is not adequately designed for long-term behavior change [92-95]. WittyFit is based on evidence-based practices. Even if there are widely varying professional backgrounds in WittyFit, we will further include behavioral components which may help the potential long-term development and evolution of WittyFit and which would also help to identify additional intervention targets. The findings from WittyFit should be significant for practical use in public health, especially as software and mobile apps are being increasingly used in health interventions [96]. The cost up until now to design WittyFit has been €1 million. Investments are still ongoing (and will continue) for a continuous improvement of WittyFit. This amount of money may seem huge, but dissemination of WittyFit within companies and contracts permits us to have an equilibrate business model (WittyFit was created in 2014). The challenging target will be to build a personal health system for workers with the capacity to interact with different physical activity and stress levels, sleep, or other health-related objectives using a computing system such as the one we are developing [73]. We will have the possibility to register a license and develop industrial applications.

### Conclusion

In conclusion, the name WittyFit came from Witty and Fitness. The concept of WittyFit reflects the concept of health from the World Health Organization: being spiritually and physically healthy. WittyFit is a health-monitoring, health-promoting initiative that may improve the health of workers and health of companies. WittyFit will evolve with the waves of connected objects further increasing its data accuracy with objective measures. WittyFit may constitute a powerful epidemiological database. Finally, the WittyFit concept may be spread in the general population and be a generalizable useful tool for prevention.

## Appendix

Multimedia Appendix 1 Original protocol.

## Abbreviations

ES: effect size
