# Supplementary material for: WittyFit—Live Your Work Differently: Study Protocol for a Workplace-Delivered Health Promotion
Source: JMIR Res Protoc. 2017 Apr 13;6(4):e58. doi: 10.2196/resprot.6267 (PMC5408138; doi:10.2196/resprot.6267)

WittyFit
Live your work differently

Brief title: WittyFit

http://www.wittyfit.com

Version: 1 date: November 25th, 2014

Administering officer:

CHU Clermont-Ferrand
58 rue Montalembert
63003 Clermont-Ferrand cedex 1
Clinical research and innovation direction
Tél : 04.73.751.195 / Fax : 04.73.754.730

Investigator coordinator:

Dr **Frédéric Dutheil**, MD, PhD, fred_dutheil@yahoo.fr, +33 6 88 22 48 48, fax: +33 4 73 27 46 49
Preventive and Occupational medicine, University Hospital of Clermont-Ferrand (CHU), France
Laboratory of Metabolic Adaptations to Exercise in Physiological and Pathological conditions (AME2P, EA 3533), Blaise Pascal University, Clermont-Ferrand, France
School of Exercise Science, Faculty of Health Science, Australian Catholic University, Melbourne, Australia; www.acu.edu.au

Collaborators:

- **Thomas Cornet**, thomas.cornet@wittyfit.com, +33 6 14 55 82 42
  Director General of WITTYFIT®, 4 avenue Laurent Cély, Asnières-sur-Seine
- **Samuel Dewavrin**, samuel.dewavrin@wittyfit.com, +33 6 86 00 21 88
  President of WITTYFIT®, 4 avenue Laurent Cély, Asnières-sur-Seine
- Pr **Martine Duclos**, MD, PhD, mduclos@chu-clermontferrand.fr, +33 4 73 751 564, fax: +33 4 73 75 16 61; Director of Sports Medicine, University Hospital of Clermont-Ferrand (CHU), France
  Pr **Geraldine Naughton**, PhD, geraldine.naughton@acu.edu.au, +61 3 9953 3034, School of Exercise Science, Faculty of Health Science, Australian Catholic University, Melbourne, Australia; www.acu.edu.au
- Dr **Jean-Claude Chatard**, MD, PhD, chatard@univ-st-etienne.fr; +33 4 77 42 72 34, fax: +33 4 77 12 72 29,Exercise Physiology, University Hospital of Saint-Etienne, France

Methodologist:

Dr **Bruno Pereira**, PhD, bpereira@chu-clermontferrand.fr, +33 4 73 75 49 64
The Clinical Research and Innovation Direction, CHU Clermont-Ferrand, France

Abstract

Title: WittyFit – Live your work differently

Keywords: health, work, lifestyle, behavior, management, stress, physical activity, nutrition, mortality, morbidity, public health

Administering officer: University Hospital of Clermont-Ferrand (CHU)

Research Team: **Frédéric Dutheil,** CHU Clermont-Ferrand, France; **Thomas Cornet**, **Samuel Dewavrin**, WittyFit®, Asnières, France; **Martine Duclos**, CHU Clermont-Ferrand, France; **Geraldine Naughton**, ACU, Australia; **Jean-Claude Chatard**, CHU Saint-Etienne, France

Methodologist: **Bruno Pereira**, CHU Clermont-Ferrand, France

Type of studies: observational

Background:
We spend one third of our life working. Age of retirement is regularly pushed back. The main challenge in the nearest future will be to maintain workers healthy to deal with their work till retirement. Morbidity before retirement has a huge cost, both in public health and economically for companies. Numerous factors increase morbidity such as stress at work, sedentary and low physical activity, and poor nutrition habits. Nowadays, digital world wildly invades our lives and offer a no-limit possibility to interact with individuals, everywhere. Thus, a software able to understand an individual in its globality seems to address this challenge. Moreover, managers need to understand the actions needed within their company.

Hypothesis: WittyFit will increase life expectancy and well-being.

Objectives: The objective of WittyFit is to increase life expectancy and well-being. Wittyfit will decrease premature mortality and morbidity. WittyFit will constitute the most powerful database of the world to build strong evidence and new knowledge on the relationships between work, behavior, and health, based on a large amount of epidemiological data.

Number of subjects needed: 100 000 followed over 5 years regarding life expectancy. However, the study will be unlimited regarding its epidemiological design.

Selection criteria: none, all workers who want to participate to the WittyFit concept will be included.

Number of centers for this study: 1
Medical data will be kept by the CHU Clermont-Ferrand, France.

Method: 1) Whereas other interventional software focus on a specific aim (relaxation, musculo-skelettal disorder, etc), Wittyfit® has the ambition to promote health with a global understanding of workers, based on continuous and updated scientific knowledge (evidence based medicine). A feedback is given on targets screened from questionnaires through e-learning and personalized motivating messages. 2) The second novelty of WittyFit is that managers will also have an anonymous feedback on the general state of health and problems encountered by its employees, in general or by department if the sample size is sufficiently high. Managers will indeed be able to target specific actions such as promoting physical activity at work or helping its employees to quit smoking. 3) Third, WittyFit is a prevention tool in collaboration with occupational medicine, which may help to detect a high risk toward the integrity of an employee. 4) Moreover, WittyFit is in fact based on two databases: "WittyFit" which deals with behavioral data, and "WittyFit Research" which deals with medical data. To guarantee the highest level of security, the two databases are separate and do not communicate together; physicians from the CHU of Clermont-Ferrand will be the only individuals with an access to medical data. 5) Finally, WittyFit may constitute the most powerful database of the world to build strong evidence and new knowledge on the relationships between lifestyle and health, based on huge amount of epidemiological data. 6) At last, WittyFit will evolve with the waves of connected objects further increasing its data accuracy, with the use of devices such as pedometers, heart rate monitors, accelerometers, thermometer, etc. 7) WittyFit is building a rainbow: work (shiftwork, sedentary job, particular occupations, etc) / psychology and physiology / statistics modeling / public health, and in general, health.

Parameters measured: Well-being regarding 3 main categories: "Physically", "Mentally", "At Work"; each divided in subcategories: "Physically" explores Nutrition, Activity, Sleep, Musculo-skelettal; "Mentally" explores Stress and Mood; "At Work" explores job strain, latitude decision, work organization and tasks, social support, and recognition. The validated questionnaires allow us to explore the individual and his environment in its globality: Karasek, Sieigrist, Pittsburg, Hospital and Anxiety Depression Scale, all the nutrition parameters (from kcal to each mineral, each aminoacid and each fatty acid), etc. All medical data are only accessible from WittyFit Research (medical history, treatments, etc). All data are anonymous and the name of the employee is absolutely never implemented in the database, at any time. The database is implemented from the human resource (HR) number, which is moreover automatically converted in another number on the WittyFit database. When a number disappear, the company will provide the information of retirement, change for another company, or death (premature mortality). After his retirement, an employee will continue to use WittyFit if he wishes, as well as a non-worker.

Benefits and adverse effects: Benefits are an increased life expectancy and well-being. Adverse effects are improbably to occur.

Measures to reduce or avoid bias: Large sample size; Generalizability; Worker de-identification to blind assessors of participant status when processing data.

Duration: unlimited. However, data will be extensively studied in order to product a considerable amount of advanced knowledge.

Funding: WittyFit is a private-public partnership with no-cost for the CHU Clermont-Ferrand. WittyFit provides the data. The CHU provides its expertise and publishes in high impact factor journals.

Résumé

Titre: WittyFit – Vivez votre travail autrement

Mots-clés: santé, travail, mode de vie, comportements, management, stress, activité physique, nutrition, mortalité, morbidité, santé publique

Institution administrative: CHU Clermont-Ferrand

Equipe de recherche: **Frédéric Dutheil,** CHU Clermont-Ferrand, France; **Thomas Cornet**, **Samuel Dewavrin**, WittyFit®, Asnières, France; **Martine Duclos**, CHU Clermont-Ferrand, France; **Geraldine Naughton**, ACU, Australia; **Jean-Claude Chatard**, CHU Saint-Etienne, France

Méthodologiste: **Bruno Pereira**, CHU Clermont-Ferrand, France

Type d'étude: observationnelle

Contexte: Nous passons un tiers de notre vie au travail. L'âge du départ à la retraite est régulièrement repoussé. Le principal défi dans un proche avenir sera de maintenir les travailleurs en bonne santé jusqu'à la retraite. La morbidité avant la retraite a un coût majeur, à la fois en termes de santé publique et au niveau économique pour les entreprises. De nombreux facteurs augmentent la morbidité, tels que le stress au travail, la sédentarité et une faible activité physique, ou de mauvaises habitudes alimentaires. Le monde numérique envahit notre environnement et offre une possibilité sans limite pour interagir avec des personnes, partout dans le monde. Ainsi, un logiciel capable de comprendre un individu dans sa globalité semble répondre à ce défi. En outre, les dirigeants doivent pouvoir connaître les actions cibles prioritaires à mener au sein de leur entreprise.

Hypothèse: WittyFit augmentera l'espérance de vie et le bien être

Objectifs: L'objectif de WittyFit est d'augmenter l'espérance de vie et le bien-être. Wittyfit diminuera la mortalité et la morbidité prématurées. WittyFit constituera la base de données la plus complète, au niveau international, pour établir des preuves solides entre les relations travail, comportement, santé, grâce à un suivi épidémiologique de masse.

Nombre de participants nécessaires: 100 000 suivis sur 5 ans en ce qui concerne l'espérance de vie. Cependant, l'étude sera illimitée au regard de son caractère épidémiologique.

Critères d'inclusion: aucun, tous les travailleurs qui veulent participer au concept WittyFit seront inclus.

Nombre de centres pour cette étude: 1
Les données médicales seront conservées par le CHU Clermont-Ferrand, France.

Méthode: 1) Alors que les autres logiciels interventionnels se focalisent sur un objectif spécifique (relaxation, troubles musculo-squelettiques, etc.), Wittyfit® a pour ambition de promouvoir la santé avec une compréhension globale des travailleurs, en se fondant sur la médecine basée sur des preuves (Evidence Based Medicine - EBM). Un retour personnalisé est donné au salarié sur ses réponses aux questionnaires, avec ses objectifs individuels, grâce à des formations en e-learning et des messages personnalisés de motivation/information. 2) La seconde nouveauté de WittyFit est que les dirigeants de l'entreprise auront également un retour anonyme sur l'état général de santé et les problèmes rencontrés par ses employés, en général ou par département si la taille de celui-ci est suffisamment élevée. Les dirigeants pourront ainsi être en mesure de cibler des actions spécifiques telles que la promotion de l'activité physique au travail ou l'aide au sevrage tabagique. 3) Troisièmement, WittyFit est un outil de prévention en collaboration avec la médecine du travail, ce qui peut être une aide dans la gestion d'un risque concernant l'intégrité d'un employé. 4) En outre, WittyFit est composé de deux bases de données: "WittyFit" qui traite des données comportementales, et "WittyFit recherche" qui traite des données médicales. Afin de garantir le plus haut niveau de sécurité, les deux bases de données sont séparés et ne communiquent pas entre elles; les médecins du CHU de Clermont-Ferrand seront les seules personnes ayant un accès aux données médicales. 5) Enfin, WittyFit a pour vocation de constituer une base de données épidémiologiques mondiale de grande envergure pour apporter des preuves solides et de nouvelles connaissances sur les relations entre mode de vie et santé. 6) Enfin, WittyFit évoluera avec les vagues d'objets interconnectés pour augmenter encore la précision de ses données, avec l'utilisation de dispositifs tels que podomètres, moniteurs de fréquence cardiaque, accéléromètres, thermomètre, etc. 7) WittyFit construit un arc en ciel entre: travail (travail posté, travail sédentaire, professions, etc.) / psychologie et physiologie / statistiques et modélisation / santé publique, et en général, la santé.

Paramètres mesurés: Le bien-être au travers trois catégories principales: «physiquement», «mentalement», «au travail»; chaque catégorie est divisée en sous-catégories: "Physiquement" explore nutrition, activité physique, sommeil, troubles musculo-squelettiques; «Mentalement" explore le stress et l'humeur; «Au travail» explore le stress au travail, la latitude décisionnelle, l'organisation du travail et des tâches, le soutien social et la reconnaissance. Les questionnaires validés nous permettront d'explorer l'individu et son environnement dans sa globalité: Karasek, Sieigrist, Pittsburg, échelle anxiété-dépression (HAD), tous les paramètres de la nutrition (des kcal aux minéraux, en passant par les acides aminés et la part de chaque acide gras), etc. Les données médicales ne seront accessibles qu'à partir WittyFit Research® (antécédents médicaux, traitements, etc.). Toutes les données seront anonymes et le nom de l'employé ne sera absolument jamais présent dans la base de données, à aucun moment. La base de données est mise en œuvre à partir du numéro RH (ressources humaines), qui est en plus automatiquement converti en un autre numéro sur la base de données WittyFit®. Quand un salarié n'est plus présent dans l'entreprise, celle-ci fournira les informations suivantes: départ à la retraite, départ pour une autre société, décès. Le salarié pourra continuer à bénéficier de WittyFit s'il le souhaite à son départ en retraite, comme un non salarié.

Bénéfices et effets indésirables: Les avantages sont une espérance de vie et un bien-être accrus. Les effets indésirables sont improbables.

Mesures pour réduire les biais: Nombre de sujets important; Généralisabilité; Anonymisation pour masquer le statut des participants lors de l'analyse des données.

Durée: illimité. Toutefois, les données seront continuellement exploitées afin d'offrir fréquemment une production scientifique de qualité.

Financement: WittyFit est un partenariat public-privé sans coût pour le CHU de Clermont-Ferrand. WittyFit fournit les données. Le CHU apporte son expertise et publie dans des revues de fort niveau de preuve.

Summary

1. General informations ______ *10*

1.1. Title ______ 10

1.2. Promotor code ______ Erreur ! Signet non défini.

1.3. Promotor ______ 10

1.4. Coordination and follow-up ______ Erreur ! Signet non défini.

1.5. Investigators ______ 10

1.5.1. Investigator coordinator ______ 10

1.5.2. Collaborators ______ 10

1.6. Methodologist ______ 10

1.7. Places of study completion ______ 11

1.8. Places where the data will be treated ______ 11

1.9. Ethics committee ______ 11

1.10. Timetable ______ 11

2. Background ______ *11*

2.1. A main challenge ______ 11

2.2. Impact of work stress, diet and exercise ______ 12

2.2.1. Stress at work ______ 12

2.2.2. Diet ______ 12

2.2.3. Physical activity ______ 12

2.3. Hypothesis ______ 13

2.4. Benefits and adverse effects ______ 13

2.5. Expected long-term impacts ______ 13

3. Objectives ______ *13*

3.1. Primary objective ______ 13

3.2. Secondary objectives ______ 13

4. Description of the study ______ *14*

4.1. Type of the study ______ Erreur ! Signet non défini.

4.2. Category of research ______ Erreur ! Signet non défini.

5. Participants ______ *14*

5.1. Inclusion criteria ______ 14

5.2. Exclusion criteria ______ 14

5.3. Premature shutdown of the research ______ 14

5.4. Period of exclusion and participation in another research ______ 14

5.5. Compensation of volunteers ______ 14

5.6. Recruitment procedures and Number of centers for this study ______ 14

5.7. Duration of participation per individual ______ 14

6. Outcomes and Parameters measured ______ *15*

6.1. Main outcome 15
6.2. Secondary outcomes 15
6.3. Time of measurements 15
7. Method *16*
7.1. A global understanding of workers 16
7.2. A manager feedback 16
7.2.1. The Middle Management 17
7.2.2. The Top Management 17
7.3. Collaborate with Occupational medicine 17
7.4. WittyFit Research 18
7.5. Epidemiological database 18
7.6. Connected objects 18
7.7. Building a rainbow 18
8. Statistics *18*
8.1. Number of subjects needed 18
8.2. Generalities on analyses 19
8.3. Primary analyses 19
8.4. Secondary analyses 19
8.5. Method taking into account missing, unused or invalid data 20
8.6. Statistician 20
8.7. Measures to reduce or avoid bias 20
9. Security Assessment - Management of adverse events *Erreur ! Signet non défini.*
9.1. Definitions Erreur ! Signet non défini.
9.2. Description of the parameters of safety assessment Erreur ! Signet non défini.
9.3. Reporting of serious adverse events Erreur ! Signet non défini.
9.4. Adverse effects Erreur ! Signet non défini.
9.5. Monitoring of subjects with an adverse event Erreur ! Signet non défini.
10. Access rights to source data and documents *Erreur ! Signet non défini.*
10.1. Data access Erreur ! Signet non défini.
10.2. Source data Erreur ! Signet non défini.
10.3. Privacy policy Erreur ! Signet non défini.
11. Quality control and assurance *Erreur ! Signet non défini.*
11.1. Commitment of investigators and the promoter Erreur ! Signet non défini.
11.2. Quality assurance Erreur ! Signet non défini.
11.3. Quality control Erreur ! Signet non défini.
11.4. Case Report Form Erreur ! Signet non défini.
12. Ethics *20*
12.1. Ethics Committee 20

12.2. Information letter and consent form ____ 21
12.3. Protocol amendments ____ 21
12.4. Support for research ____ 21
13. Data processing and storage of documents and data related to research ____ 21
13.1. Collection and processing of data ____ 21
13.2. CNIL ____ 21
13.3. Digital Preservation ____ 21
14. Costs, Funding and Insurances ____ 22
14.1. Costs of the study ____ Erreur ! Signet non défini.
14.2. Insurances ____ Erreur ! Signet non défini.
15. Communication and publishing rules ____ 22
16. Feasibility of this study ____ 22
17. References ____ 23
18. Summary for annex ____ 25

1. General informations

1.1. Title

Title: WittyFit – Live your work differently

1.2. Administrative officer

University Hospital CHU Clermont-Ferrand
58 rue Montalembert
63003 Clermont-Ferrand cedex 1
Clinical research and innovation direction
Tél : 04.73.751.195 / Fax : 04.73.754.730

1.3. Investigators

1.3.1. Investigator coordinator

Dr **Frédéric Dutheil**, MD, PhD, fred_dutheil@yahoo.fr, +33 6 88 22 48 48, fax: +33 4 73 27 46 49
Preventive and Occupational medicine, University Hospital of Clermont-Ferrand (CHU), France
Laboratory of Metabolic Adaptations to Exercise in Physiological and Pathological conditions (AME2P, EA 3533), Blaise Pascal University, Clermont-Ferrand, France
School of Exercise Science, Faculty of Health Science, Australian Catholic University, Melbourne, Australia; www.acu.edu.au

1.3.2. Collaborators

- **Thomas Cornet**, thomas.cornet@wittyfit.com, +33 6 14 55 82 42
  Director General of WITTYFIT®, 4 avenue Laurent Cély, Asnières-sur-Seine
- **Samuel Dewavrin**, samuel.dewavrin@wittyfit.com, +33 6 86 00 21 88
  President of WITTYFIT®, 4 avenue Laurent Cély, Asnières-sur-Seine
- Pr **Martine Duclos**, MD, PhD, mduclos@chu-clermontferrand.fr, +33 4 73 751 564, fax: +33 4 73 75 16 61; Director of Sports Medicine, University Hospital of Clermont-Ferrand (CHU), France
- Pr **Geraldine Naughton**, PhD, geraldine.naughton@acu.edu.au, +61 3 9953 3034, School of Exercise Science, Faculty of Health Science, Australian Catholic University, Melbourne, Australia; www.acu.edu.au
- Dr **Jean-Claude Chatard**, MD, PhD, chatard@univ-st-etienne.fr; +33 4 77 42 72 34, fax: +33 4 77 12 72 29,Exercise Physiology, University Hospital of Saint-Etienne, France

1.4. Methodologist

Dr **Bruno Pereira**, PhD, bpereira@chu-clermontferrand.fr, +33 4 73 75 49 64
The Clinical Research and Innovation Direction, CHU Clermont-Ferrand, France

1.5. Places of study completion

Any individual throughout the world.

1.6. Places where the data will be treated

Data will be analyzed at The Clinical Research and Innovation Direction, CHU Clermont-Ferrand, France.

1.7. Ethics committee

Comité de Protection des Personnes Sud Est VI
CHU de Clermont-Ferrand, 58 rue Montalembert 63000 Clermont-Ferrand, France

1.8. Timetable

Ethics: February 2015
Beginning of the protocol: February 2015
Ending of the protocol: no ending
Publications: from 2015

2. Background

2.1. A main challenge

We spend one third of our life working. Age of retirement is regularly pushed back.[1] The main challenge in the nearest future will be to maintain workers healthy to deal with their work till retirement.[1] Morbidity before retirement has a huge cost, both in public health and economically for companies. Numerous factors increase morbidity such as stress at work,[2] sedentary and low physical activity,[3] and poor nutrition habits.[4-7] Nowadays, digital world wildly invades our lives and offer a no-limit possibility to interact with individuals, everywhere. Thus, a software able to understand an individual in its globality seems to address this challenge. Moreover, managers need to understand the actions needed within their company.

2.2. Impact of work stress, diet and exercise

2.2.1. Stress at work

Working conditions are a strong determinants of morbidity.[1,8-21] For example, a low social support at work is linked with cardiovascular events[22] and depression[23]. Moreover, there is a deleterious contagious effects of poor psychological working conditions such as emotional exhaustion and depersonalization.[24] Changes in organization, conflict of loyalties resulting from work change may also lead to suicide.[25] Stress management programs taking into account specific work organization could be implemented at the worksite, with possible preventive advantages.[26] A global understanding of individual perception is needed to manage stress at work.[27] Eventually, stress at work is a complex interplay with sleep,[28] diet[29] and physical activity.[30,31]

2.2.2. Diet

There are numerous and extensive literature on benefits of a healthy diet. Nearly all aspects of food intake and health have been investigated: from the influence of the breakfast[4] to the influence of dietary sugars[6] or coffee[5] on heart disease. Some working conditions, such as shift work, are associated with abnormal eating behavior such as alterations to timing of meals and type of food eaten,[32] promoting an increased risk of developing obesity and metabolic disorders.[33] In addition, stress also promotes unhealthy eating behaviors and development of obesity.[34] However, those studies were mainly cross-sectional. Interventional studies on a huge number of participants remain scarce. Moreover, WittyFit should provide the first interventional study with an epidemiological cohort frame including specific details on working conditions. This is in agreement with recommendations promoting more health preventive initiatives focusing on dietary counseling,[32] particularly on individuals more exposed to stress.[35]

2.2.3. Physical activity

The American College of Sports Medicine recommends a minimum of 150 minutes of moderate, or 75 minutes of vigorous intensity of physical activity per week to achieve and maintain global health for adults, and at least 30 minutes of moderate intensity a day.[36] Moreover, even smaller amount,[37] and even only standing[3] without further physical activity have shown benefits on life-expectancy. However, the more physical activity is done, the longer life expectancy and health benefits are obtained, even if sedentary individuals start to train at a high level after 50 years of age.[38] Therefore, any form of physical activity is better than no activity,[3,36,37] and it is never too late to start exercising.[38]

The benefits of physical activity on mental health have been well documented.[39] Along with reducing workplace absenteeism,[39,40] a strong negative association exists between physical activity and mood states.[41] This is highlighted by the broad public opinion that physical activity is an effective coping mechanism for stress.[42]

2.3. Hypothesis

We hypothesize that WittyFit will increase life expectancy and well-being.

2.4. Benefits and adverse effects

Benefits: Benefits are an increased life expectancy and well-being.
Adverse effects: Adverse effects are improbably to occur.

2.5. Expected long-term impacts

Both collectively and for the participants, WittyFit will change the relations with work and build a different way to live our job. Expected long-term impacts are global: economically for the companies by decreasing absenteeism, work-related injuries and occupational diseases, and personally for the participants by increasing well-being and life expectancy. WittyFit could be a major public health component at work.

3. Objectives

The global aim is to build an epidemiological database combining major lifestyle parameters.

3.1. Primary objective

The objective of WittyFit is to decrease premature mortality.

3.2. Secondary objectives

WittyFit will decrease morbidity and improve well-being. WittyFit will constitute the most powerful database of the world to build strong evidence and new knowledge on the relationships between work, behavior, and health, based on a large amount of epidemiological data.

4. Description of the study: observational study

Observational epidemiological study.

5. Participants

5.1. Inclusion criteria

All workers who want to participate to the WittyFit concept will be included

5.2. Exclusion criteria

Patient's refusal to participate

5.3. Premature shutdown of the research

Intermediate analysis will be conduct several times per year and although improbable, any adverse effects will be extensively studied

5.4. Period of exclusion and participation in another research

Another protocol could be bone simultaneously.

5.5. Compensation of volunteers

No indemnity.

5.6. Recruitment procedures and Number of centers for this study

WittyFitt will deliver his process to the entire workforce of worldwide companies.
Data will be analyzed at The Clinical Research and Innovation Direction, CHU Clermont-Ferrand, France.

5.7. Duration of participation per individual

Unlimited. However, data will be extensively studied in order to product a considerable amount of advanced knowledge.

6. Outcomes and Parameters measured

6.1. Main outcome

Life expectancy

6.2. Secondary outcomes

Workers will fulfill a visual analog scale (VAS) of Well-being regarding 3 main categories: “Physically”, “Mentally”, “At Work”.

Each main category is divided in subcategories:

- “Physically” explores Nutrition, Activity, Sleep, Musculo-skelettal. Nutrition will be assessed through day-recording food intake. Physical activity will be at-work and during leisure time, as well as sedentary. Sleep will be assessed from Pittsburg questionnaire.[28] Specific questions will complete these questionnaires
- “Mentally” explores Stress and Mood, with the use of validated questionnaires such as Hospital and Anxiety Depression Scale.[43]
- “At Work” explores job strain, latitude decision, work organization and tasks, social support, and recognition. Validated questionnaires will be used, such as Karasek[15,16,44] and Sieigrist[12,17,45] questionnaires.

All medical data are only accessible from WittyFit Research (medical history, treatments, etc). All data are anonymous and the name of the employee is absolutely never implemented in the database, at any time. The database is implemented from the human resource (HR) number, which is moreover automatically converted in another number on the WittyFit database. When a number disappear, the company will provide the information of retirement, change for another company, or death (premature mortality).

6.3. Time of measurements

Every participant can fulfill questionnaires when he wants. However, participants will be asked to fulfill <u>general VAS</u> (“Physically”, “Mentally”, “At Work”) <u>every 15 days</u>; and <u>detailed questionnaires every 6 months</u>.

7. Method

7.1. A global understanding of workers

Whereas other interventional software focus on a specific aim (relaxation, musculo-skelettal disorder, etc), Wittyfit® has the ambition to promote health with a global understanding of workers, based on continuous and updated scientific knowledge (evidence based medicine). A feedback is given on targets screened from questionnaires through e-learning or “did you know” informations, based on personalized analyses of questionnaires.

Each E-learning module is based on a 3 steps approach:

- First, answer a quiz (pretest)
- Second, understand the issue,
- Third, act on the issue,
- Fourth, answer a quiz.

The employee has access to his own personal data on a panel of indicators displaying his progress and his success. The employee may also input new ideas in the “Digital Idea Box” and “likes” ideas of others. He may participate to poles issued by the company regarding evolution and changes. When actions are needed, a red dot will appear on the access button of the relevant topic.

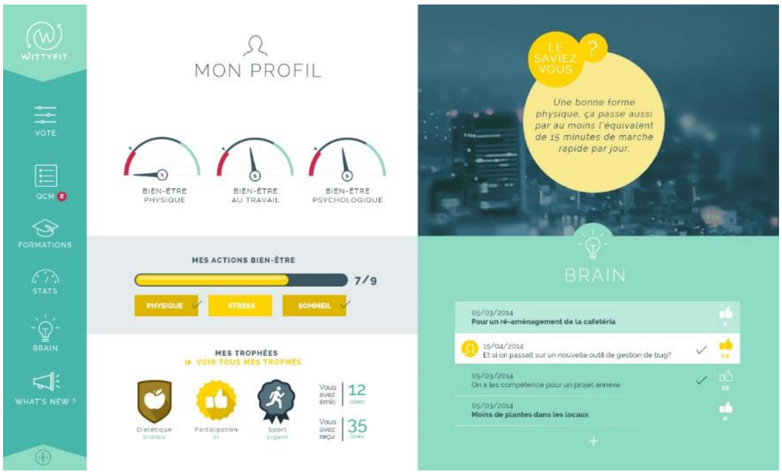

7.2. A manager feedback

The second novelty of WittyFit is that managers will also have an anonymous feedback on the general state of health and problems encountered by its employees, in general or by department if the sample size is sufficiently high. Managers will indeed be able to target specific actions such as promoting physical activity at work or helping its employees to quit smoking.

7.2.1. The Middle Management

- Has access to his department average for each main VAS (“Physical”, “Mental” and “At work”) and subcategories of “At Work”
- Gets warning on a low average VAS showing a weakness of his team in a specific category
- Gets feedback of the relevant issues in his department
- Has access to a best practice database
- Has access to the Forum Manager anonymous community to share experience
- Has a Go/No Go functionality regarding the proposals in the “Digital Idea Box”. If not in position to decide, the middle management can promote an idea for the top management

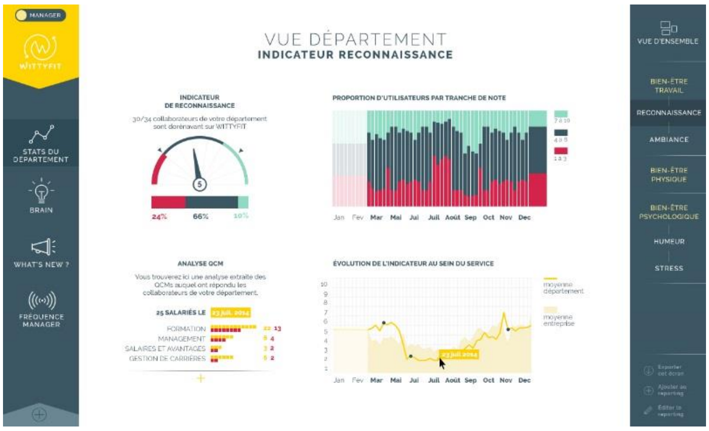

7.2.2. The Top Management

- Real time and historical access to average VAS results of “Physical”, “Mental” and “At work” indicators and subcategories of “At Work” by division, location, department, age, seniority, gender, job title…
- Gets feedback of the relevant issues by division, location, department, age, seniority, gender, job title…
- Monitor the turn-over and absenteeism in regard to the VAS evolutions
- Access the “Digital Idea Box” and put in motion relevant ideas
- Use the “what’s new” editing tool (pole) to involve the work force in the changes to come.

7.3. Collaborate with Occupational medicine

- Real time and historical access to average VAS results of “Physical”, “Mental” and “At work” indicators and all subcategories by division, location, department, age, seniority, gender, job title…
- Gets feedback of the relevant issues by division, location, department, age, seniority, gender, job title…
- Monitor the turn-over and absenteeism in regard to the VAS evolutions
- No access to the “Digital Idea Box”
- No access to the “what’s new” editing tool (pole)
- **If a high risk is detected toward the integrity of an employee, he is the only one with the ability to break anonymity**.

7.4. WittyFit Research

WittyFit is in fact based on two databases: “WittyFit” which deals with behavioral data, and “WittyFit Research” which deals with medical data. To guarantee the highest level of security, the two databases are separate and do not communicate together; physicians from the CHU of Clermont-Ferrand will be the only individuals with an access to medical data.

7.5. Epidemiological database

WittyFit may constitute the most powerful database of the world to build strong evidence and new knowledge on the relationships between lifestyle and health, based on huge amount of epidemiological data

7.6. Connected objects

At last, WittyFit will evolve with the waves of connected objects further increasing its data accuracy, with the use of devices such as pedometers, heart rate monitors, accelerometers, thermometer, etc

7.7. Building a rainbow

WittyFit is building a rainbow: work (shiftwork, sedentary job, particular occupations, etc) / psychology and physiology / statistics modeling / public health, and in general, health

8. Statistics

8.1. Number of subjects needed

100 000 followed over 5 years regarding life expectancy. However, the study will be unlimited regarding its epidemiological design. Some precisions could be proposed according to previous works[46] studying leisure time spent sitting in relation to total mortality in a prospective cohort of US adults[3] and job strain among blue-collar and white-collar employees as a determinant of total mortality with a 28-year population-based follow-up.[47]

Also, to study the aim objective to decrease premature mortality, statistical power estimation bad on Hazard Ratio used for censored data (HR among 1.25 and 2) and according to the literature data described previously (HR) was be proposed for type-I error of 5% (two-sided) and statistical power equals 95%.

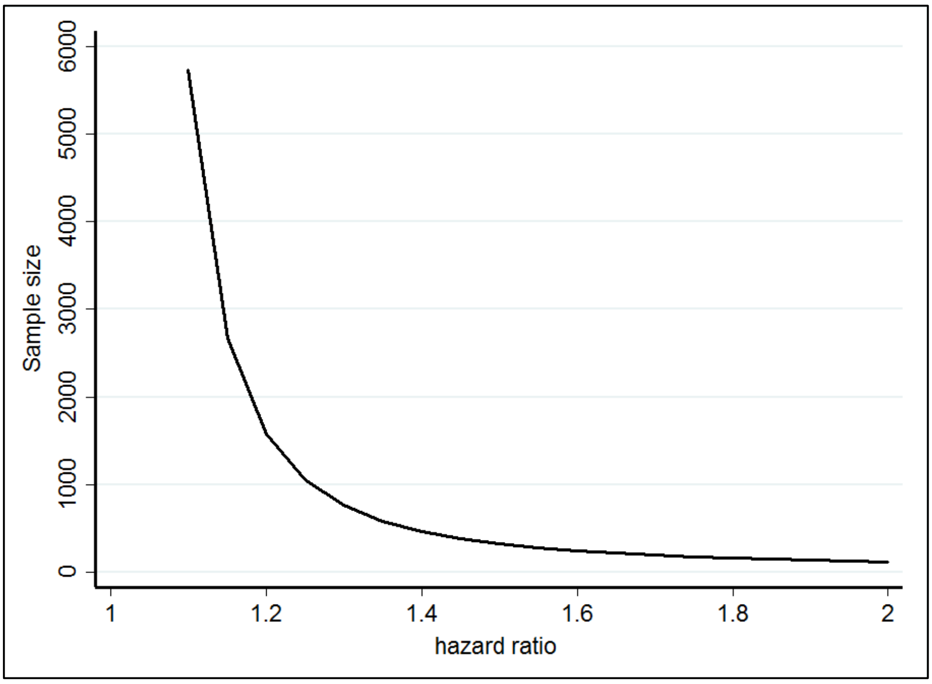

8.2. Generalities on analyses

Statistical analysis will be performed using Stata software (version 13; Stata-Corp, College Station, Tex., USA). All statistical tests will be bilateral and a $p < 0.05$ will be considered significant. Qualitative variables will be described in terms of numbers and proportions. Quantitative variables will be described in terms of numbers, average, median, standard deviation, and range. Graphic representations will be complete presentations of results.

8.3. Primary analyses

The aim objective of this protocol is to decrease premature mortality. Considering this censored data, estimation will be performed using Kaplan-Meier. Comparison with historical cohorts and previous works will be realized considering 95% confidence interval. WittyFit's effect will be also study considering log-rank comparisons and Cox proportional hazards model. The multivariate model will be considered according to statistical relevance (univariate results) and clinical/epidemiological relevance.

8.4. Secondary analyses

To study WittyFit's effect on morbidity and well-being, analyses considered on one hand comparisons with historical cohorts and literature and on other hand comparisons in paired situation in which each subject will be his own witness. To study longitudinal evolution of parameters

associated to morbidity and well-being, models for repeated data will be performed using random-effects model (linear or generalized linear) to study fixed effects and taking into account within and between subject variability.
When appropriate, comparisons between subgroups will be proposed. Usual statistical tests will be applied: Chi-squared or Fisher exact tests for comparisons between groups for categorical parameters and anova or Kruskal-Wallis test for quantitative variables. For censored data, comparisons were performed like aim analysis by log-rank test and Cox proportional hazards model. Considering repeated correlated data, marginal models may be preferred.

8.5. Method taking into account missing, unused or invalid data

A sensitivity analysis of missing data will be considered in order to assess the level of attrition and to characterize the statistical nature (Missing At Random, Missing Completely At Random, Not Missing At Random) to propose the method of imputation most appropriate.[48-50]

8.6. Statistician

Dr **Bruno Pereira**, CHU Clermont-Ferrand, France

8.7. Measures to reduce or avoid bias

- Worker de-identification to blind assessors of participant status when processing data
- Large sample size
- Generalizability

9. Ethics

9.1. Ethics Committee

The protocol, information and consent form as well as the CRF for the study will be submitted for review to the Ethics Committee of Clermont-Ferrand (South-East VI) – Comité de Protection des Personnes Sud Est VI.
Notification of the approval of the Ethics Committee will be sent to the study promotor and the competent authority. An application for authorization shall be sent by the Promoter to the competent authority before the start of the study.

9.2. Information letter and consent form

Patients should be informed fully and fairly, in understandable terms, of the objectives and constraints of the study, possible risks, monitoring measures, and of their rights to refuse to participate in the study or the opportunity to withdraw at any time. The investigator must also inform the individuals of the approval of the ethics committee.
All these informations are in the message written on the website when entering WittyFit Research.

9.3. Protocol amendments

Changes to the protocol will be considered as substantial or not. They will, by their nature, subject to a new opinion of the Ethics Committee and/or permission of the competent authority.

9.4. Support for research

This is an observational study in ecological environment, without randomization.

10. Data processing and storage of documents and data related to research

10.1. Collection and processing of data

Data will be entered by participants on WittyFit® and WIttyFit Research®.
Statistical analysis will be performed by members of the team.

10.2. CNIL

This study is part of the "Reference Methodology" (MR-001) under the provisions of the Act of 6 August 2004 on the protection of individuals with regard to the processing of personal data and amending Act of 6 January 1978 relating to computers, files and freedoms. This change was approved on January 5, 2006. CHU Clermont-Ferrand, the study sponsor, has signed a commitment to comply with the "Reference Methodology" dated 15/03/2007.
For studies not included in the scope of the "Reference Methodology" (MR-001), plan to ask the CCTIRS, then the CNIL.

10.3. Digital Preservation

The following documents will be archived by the name of the study in the offices of the occupational medicine department of the Clermont-Ferrand University Hospital until the end of the period of practical use (duration of the study + the time required for data analysis).
These documents are:

- Protocol and annexes, any amendments,
- Information forms and original signed consents
- Individual data (authenticated copies of raw data)
- Monitoring documents
- Statistical analyses
- Final report of the study

At the end of the period of practical utility, all documents to be archived, as defined in the procedure PG.06.005 "Managing documentation for protocols" of the Clermont-Ferrand University Hospital will be transferred to the central archives and will be under the responsibility of the Promoter for 15 years after the end of the study in accordance with institutional practices.
No displacement or destruction shall be made without the consent of the Promoter. After 15 years, the developer will be consulted for destruction. All data, all documents and reports will be subject to audit or inspection.

11. Costs and Funding

WittyFit is a private-public partnership with no-cost for the CHU Clermont-Ferrand. WittyFit provides the data. The CHU provides its expertise and publishes in high impact factor journals.

12. Communication and publishing rules

The data will be disclosed after prior joint agreement of the investigator and promotor. The results will be communicated and publicated. The study will be pre-registered on an open access website: Clinical trials.gov.

13. Feasibility of this study

Skills of the research team:

Dutheil F, Boudet G, Perrier C, Lac G, Ouchchane L, Chamoux A, Duclos M, Schmidt J. JOBSTRESS study: Comparison of heart rate variability in emergency physicians working a 24-hour shift or a 14-hour night shift - A randomized trial. *International Journal of Cardiology.* 2012 Jul;158(2):322-325. Impact factor 2013: **7.1**

Dutheil F, Trousselard M, Perrier C, Lac G, Chamoux A, Duclos M, Naughton G, Mnatzaganian G, Schmidt J. Urinary Interleukin-8 Is a Biomarker of Stress in Emergency Physicians, Especially with Advancing Age - The

JOBSTRESS* Randomized Trial. *PLoS One*. 2013 Aug 19;8(8):e71658. doi: 10.1371/journal.pone.0071658. Impact factor 2013: **4.09**

Duclos M, Oppert JM, Verges B, Coliche V, Gautier JF, Guezennec Y, Reach G, Strauch G; SFD diabetes and physical activity working group. Physical activity and type 2 diabetes. Recommandations of the SFD (Francophone Diabetes Society) diabetes and physical activity working group. Diabetes Metab. 2013;39(3):205-16. doi: 10.1016/j.diabet.2013.03.005. 5-year Impact factor 2013: **3.1**

Dutheil F, Lac G, Lesourd B, Chapier R, Walther G, Vinet A, Sapin V, Verney J, Ouchchane L, Duclos M, Obert P, Courteix D. Different modalities of exercise to reduce visceral fat mass and cardiovascular risk in metabolic syndrome: the RESOLVE* randomized trial. *International Journal of Cardiology*. 2013. doi:10.1016/j.ijcard.2013.05.012. Impact factor 2013: **7.1**

Wilson MG, Chatard JC, Carre F, Hamilton B, Whyte GP, Sharma S, Chalabi H.Prevalence of electrocardiographic abnormalities in West-Asian and African male athletes. *Br J Sports Med*. 2012 Apr;46(5):341-7. doi: 10.1136/bjsm.2010.082743. 5-year Impact factor 2013: **4.34**

Recruitment opportunities: 3 billions of workers globally, 18 millions in France.

14. References

1. McGonagle AK, Fisher GG, Barnes-Farrell JL, Grosch JW. Individual and Work Factors Related to Perceived Work Ability and Labor Force Outcomes. *J. Appl. Psychol.* Oct 13 2014.
2. Backe EM, Seidler A, Latza U, Rossnagel K, Schumann B. The role of psychosocial stress at work for the development of cardiovascular diseases: a systematic review. *Int. Arch. Occup. Environ. Health.* Jan 2012;85(1):67-79.
3. Patel AV, Bernstein L, Deka A, et al. Leisure time spent sitting in relation to total mortality in a prospective cohort of US adults. *Am. J. Epidemiol.* Aug 15 2010;172(4):419-429.
4. Cahill LE, Chiuve SE, Mekary RA, et al. Prospective Study of Breakfast Eating and Incident Coronary Heart Disease in a Cohort of Male US Health Professionals. *Circulation.* Jul 23 2013;128(4):337-343.
5. Ding M, Bhupathiraju SN, Satija A, van Dam RM, Hu FB. Long-term coffee consumption and risk of cardiovascular disease: a systematic review and a dose-response meta-analysis of prospective cohort studies. *Circulation.* Feb 11 2014;129(6):643-659.
6. Te Morenga LA, Howatson AJ, Jones RM, Mann J. Dietary sugars and cardiometabolic risk: systematic review and meta-analyses of randomized controlled trials of the effects on blood pressure and lipids. *Am. J. Clin. Nutr.* May 7 2014.
7. Trichopoulou A, Bamia C, Trichopoulos D. Anatomy of health effects of Mediterranean diet: Greek EPIC prospective cohort study. *BMJ.* 2009;338:b2337.
8. Cesana G, Sega R, Ferrario M, Chiodini P, Corrao G, Mancia G. Job strain and blood pressure in employed men and women: a pooled analysis of four northern italian population samples. *Psychosom. Med.* Jul-Aug 2003;65(4):558-563.
9. Chandola T, Britton A, Brunner E, et al. Work stress and coronary heart disease: what are the mechanisms? *Eur. Heart J.* Mar 2008;29(5):640-648.
10. Chikani V, Reding D, Gunderson P, McCarty CA. Psychosocial work characteristics predict cardiovascular disease risk factors and health functioning in rural women: the Wisconsin Rural Women's Health Study. *J. Rural Health.* Fall 2005;21(4):295-302.
11. Collins SM, Karasek RA, Costas K. Job strain and autonomic indices of cardiovascular disease risk. *Am. J. Ind. Med.* Sep 2005;48(3):182-193.
12. de Jonge J, Bosma H, Peter R, Siegrist J. Job strain, effort-reward imbalance and employee well-being: a large-scale cross-sectional study. *Soc. Sci. Med.* May 2000;50(9):1317-1327.
13. Evans S, Huxley P, Gately C, et al. Mental health, burnout and job satisfaction among mental health social workers in England and Wales. *Br. J. Psychiatry.* Jan 2006;188:75-80.

14. Goldstone AR, Callaghan CJ, Mackay J, Charman S, Nashef SA. Should surgeons take a break after an intraoperative death? Attitude survey and outcome evaluation. *BMJ.* Feb 14 2004;328(7436):379.

15. Karasek R, Baker D, Marxer F, Ahlbom A, Theorell T. Job decision latitude, job demands, and cardiovascular disease: a prospective study of Swedish men. *Am. J. Public Health.* Jul 1981;71(7):694-705.

16. Karasek R, Brisson C, Kawakami N, Houtman I, Bongers P, Amick B. The Job Content Questionnaire (JCQ): an instrument for internationally comparative assessments of psychosocial job characteristics. *J. Occup. Health Psychol.* Oct 1998;3(4):322-355.

17. Siegrist J. Adverse health effects of high-effort/low-reward conditions. *J. Occup. Health Psychol.* Jan 1996;1(1):27-41.

18. Johnson JV, Hall EM. Job strain, work place social support, and cardiovascular disease: a cross-sectional study of a random sample of the Swedish working population. *Am. J. Public Health.* Oct 1988;78(10):1336-1342.

19. Pisanti R, van der Doef M, Maes S, Lazzari D, Bertini M. Job characteristics, organizational conditions, and distress/well-being among Italian and Dutch nurses: a cross-national comparison. *Int. J. Nurs. Stud.* Jul 2011;48(7):829-837.

20. Pomaki G, Maes S, Ter Doest L. Work conditions and employees' self-set goals: goal processes enhance prediction of psychological distress and well-being. *Pers Soc Psychol Bull.* Jun 2004;30(6):685-694.

21. Schnall PL, Pieper C, Schwartz JE, et al. The relationship between 'job strain,' workplace diastolic blood pressure, and left ventricular mass index. Results of a case-control study. *JAMA.* Apr 11 1990;263(14):1929-1935.

22. Andre-Petersson L, Engstrom G, Hedblad B, Janzon L, Rosvall M. Social support at work and the risk of myocardial infarction and stroke in women and men. *Soc. Sci. Med.* Feb 2007;64(4):830-841.

23. Aziah BD, Rusli BN, Winn T, Naing L, Tengku MA. Risk factors of job-related depression in laboratory technicians in Hospital Universiti Sains Malaysia (HUSM) and Kementerian Kesihatan Malaysia (KKM) hospitals in Kelantan. *Southeast Asian J. Trop. Med. Public Health.* Jun 2004;35(2):468-475.

24. Bakker AB, Le Blanc PM, Schaufeli WB. Burnout contagion among intensive care nurses. *J. Adv. Nurs.* Aug 2005;51(3):276-287.

25. Brodsky CM. Suicide attributed to work. *Suicide Life. Threat. Behav.* Winter 1977;7(4):216-229.

26. Lucini D, Riva S, Pizzinelli P, Pagani M. Stress management at the worksite: reversal of symptoms profile and cardiovascular dysregulation. *Hypertension.* Feb 2007;49(2):291-297.

27. Rusli BN, Edimansyah BA, Naing L. Working conditions, self-perceived stress, anxiety, depression and quality of life: a structural equation modelling approach. *BMC Public Health.* 2008;8:48.

28. Buysse DJ, Reynolds CF, 3rd, Monk TH, Berman SR, Kupfer DJ. The Pittsburgh Sleep Quality Index: a new instrument for psychiatric practice and research. *Psychiatry Res.* May 1989;28(2):193-213.

29. Kiecolt-Glaser JK. Stress, food, and inflammation: psychoneuroimmunology and nutrition at the cutting edge. *Psychosom. Med.* May 2010;72(4):365-369.

30. Stults-Kolehmainen MA, Sinha R. The effects of stress on physical activity and exercise. *Sports Med.* Jan 2014;44(1):81-121.

31. Hansen AM, Blangsted AK, Hansen EA, Sogaard K, Sjogaard G. Physical activity, job demand-control, perceived stress-energy, and salivary cortisol in white-collar workers. *Int. Arch. Occup. Environ. Health.* Feb 2010;83(2):143-153.

32. Wong H, Wong MC, Wong SY, Lee A. The association between shift duty and abnormal eating behavior among nurses working in a major hospital: a cross-sectional study. *Int. J. Nurs. Stud.* Aug 2010;47(8):1021-1027.

33. Esquirol Y, Perret B, Ruidavets JB, et al. Shift work and cardiovascular risk factors: new knowledge from the past decade. *Arch Cardiovasc Dis.* Dec 2011;104(12):636-668.
34. Sinha R, Jastreboff AM. Stress as a Common Risk Factor for Obesity and Addiction. *Biol. Psychiatry.* Mar 26 2013.
35. Buss J. Associations between obesity and stress and shift work among nurses. *Workplace Health Saf.* Oct 2012;60(10):453-458; quiz 459.
36. Garber CE, Blissmer B, Deschenes MR, et al. American College of Sports Medicine position stand. Quantity and quality of exercise for developing and maintaining cardiorespiratory, musculoskeletal, and neuromotor fitness in apparently healthy adults: guidance for prescribing exercise. *Med. Sci. Sports Exerc.* Jul 2011;43(7):1334-1359.
37. Wen CP, Wai JP, Tsai MK, et al. Minimum amount of physical activity for reduced mortality and extended life expectancy: a prospective cohort study. *Lancet.* Oct 1 2011;378(9798):1244-1253.
38. Byberg L, Melhus H, Gedeborg R, et al. Total mortality after changes in leisure time physical activity in 50 year old men: 35 year follow-up of population based cohort. *Br. J. Sports Med.* Jul 2009;43(7):482.
39. Bhui KS, Dinos S, Stansfeld SA, White PD. A synthesis of the evidence for managing stress at work: a review of the reviews reporting on anxiety, depression, and absenteeism. *J Environ Public Health.* 2012;2012:515874.
40. Conn VS, Hafdahl AR, Cooper PS, Brown LM, Lusk SL. Meta-analysis of workplace physical activity interventions. *Am. J. Prev. Med.* Oct 2009;37(4):330-339.
41. Fox KR. The influence of physical activity on mental well-being. *Public Health Nutr.* Sep 1999;2(3A):411-418.
42. Cairney J, Kwan M, Veldhuizen S, Faulkner GE. Who Uses Exercise as a Coping Strategy for Stress? Results From a National Survey of Canadians. *J Phys Act Health.* Mar 14 2013.
43. Zigmond AS, Snaith RP. The hospital anxiety and depression scale. *Acta Psychiatr. Scand.* Jun 1983;67(6):361-370.
44. Karasek RA. Job demands, job decision latitude, and mental strain : implications for job redesign. *Adm. Sci. Q.* 1979;24:285-308.
45. Siegrist J, Klein D, Voigt KH. Linking sociological with physiological data: the model of effort-reward imbalance at work. *Acta Physiol. Scand. Suppl.* 1997;640:112-116.
46. King AC, Hekler EB, Castro CM, et al. Exercise advice by humans versus computers: maintenance effects at 18 months. *Health Psychol.* Feb 2014;33(2):192-196.
47. von Bonsdorff MB, Seitsamo J, von Bonsdorff ME, Ilmarinen J, Nygard CH, Rantanen T. Job strain among blue-collar and white-collar employees as a determinant of total mortality: a 28-year population-based follow-up. *BMJ Open.* 2012;2(2):e000860.
48. Vangeneugden T, Molenberghs G, Laenen A, Alonso A, Geys H. Generalizability in nongaussian longitudinal clinical trial data based on generalized linear mixed models. *J. Biopharm. Stat.* 2008;18(4):691-712.
49. Zhang D. Generalized linear mixed models with varying coefficients for longitudinal data. *Biometrics.* Mar 2004;60(1):8-15.
50. Pourahmadi M, Daniels MJ. Dynamic conditionally linear mixed models for longitudinal data. *Biometrics.* Mar 2002;58(1):225-231.

15. Summary for annex

Annex 1 – Information given when entering WittyFit Research

Annex 2 – Curriculum Vitae

ANNEX 1 – Information given when entering WittyFit Research

Vous entrez dans WittyFit Research®.

WittyFit Research a pour but de collecter des données médicales afin de les mettre en relation avec les questions auxquelles vous avez répondu, et d'ainsi pouvoir répondre à différentes questions, comme par exemple les relations entre les rythmes de travail et les pathologies cardiovasculaires, l'effet de la lutte contre la sédentarité au travail sur votre état de santé… les questions sont multiples.

Ce projet a obtenu l'accord du comité d'éthique Sud Est VI le …./…./….. Le CHU de Clermont-Ferrand conservera les données sur un site distinct. L'anonymat est garanti.

ANNEX 2 – Short Curriculum Vitae of investigators

Name: Frédéric DUTHEIL

Functions: university hospital practitioner, physician in occupational medicine

Titles: MD, PhD

Establishment:
- Occupational medicine, CHU Clermont-Ferrand, Hôpital Gabriel Montpied, 58 rue Montalembert – BP 69, 63003 Clermont-Ferrand cedex

Research lab:

INSERM ☐ CNRS ☐ Others x

Address:
- School of Exercise Science (Pr G Naughton), Australian Catholic University, Locked Bag 4115 Fitzroy MDC VIC 3065, Melbourne, Australia
- Laboratory of Metabolic Adapatations to Exercise in Physiological and Pathological conditions (AME2P, EA 3533, Pr N Boisseau), Aubière, France

Phone: *+33* 6 88 22 48 48 ***Fax***: +33 4 73 27 46 49

e-mail: fred_dutheil@yahoo.fr

n° d'inscription au conseil de l'ordre: 63/5926 **n° ADELI**: 63 10 5926 8

n°RPPS: 10 100 16 14 38

Mains publications:

Ollier M, Garcier JM, Naughton G, Chamoux A, Pereira B, Dutheil F. CT scan procedure for lung cancer screening in asbestos-exposed workers. Chest. 2014 Aug.5-year impact factor 2013: **6.42**

Ollier M, Chamoux A, Naughton G, Pereira B, Dutheil F. Chest computed tomography screening for lung cancer in asbestos occupational exposure: a systematic review and meta-analysis. *Chest*. 2014 Jan 30. doi: 10.1378/chest.13-2181. 5-year impact factor 2013: **6.42**

Dutheil F, Lac G, Lesourd B, Chapier R, Walther G, Vinet A, Sapin V, Verney J, Ouchchane L, Duclos M, Obert P, Courteix D. Different modalities of exercise to reduce visceral fat mass and cardiovascular risk in metabolic syndrome: the RESOLVE* randomized trial. *International Journal of Cardiology*. 2013. doi:10.1016/j.ijcard.2013.05.012. Impact factor 2013: **7.1**

Dutheil F, Boudet G, Perrier C, Lac G, Ouchchane L, Chamoux A, Duclos M, Schmidt J. JOBSTRESS study: Comparison of heart rate variability in emergency physicians working a 24-hour shift or a 14-hour night shift - A randomized trial. *International Journal of Cardiology.* 2012 Jul;158(2):322-325. Impact factor 2013: **7.1**

Dutheil F, Trousselard M, Perrier C, Lac G, Chamoux A, Duclos M, Naughton G, Mnatzaganian G, Schmidt J. Urinary Interleukin-8 Is a Biomarker of Stress in Emergency Physicians, Especially with Advancing Age - The JOBSTRESS* Randomized Trial. *PLoS One*. 2013 Aug 19;8(8):e71658. doi: 10.1371/journal.pone.0071658. Impact factor 2013: **4.09**

November 16th, 2014:

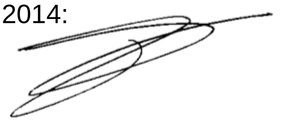

Name: Thomas CORNET

Functions: Director general of WittyFit

Titles:

Establishment:
- WITTYFIT®, 4 avenue Laurent Cély, Asnières-sur-Seine

Research lab:
INSERM ☐ CNRS ☐ Others ☐
Address:

Phone: +33 6 14 55 82 42 ***Fax***:

e-mail: thomas.cornet@wittyfit.com

n° d'inscription au conseil de l'ordre: - **n° ADELI**: -

n°RPPS: -

Mains publications:

November 16th, 2014:

Name: Samuel DEWAVRIN

Functions: President of WittyFit

Titles:

Establishment:
- WITTYFIT®, 4 avenue Laurent Cély, Asnières-sur-Seine

Research lab:

INSERM ☐ CNRS ☐ Others ☐

Address:

Phone: +33 6 14 55 82 42 ***Fax***:

e-mail: samuel.dewavrin@wittyfit.com

n° d'inscription au conseil de l'ordre: - **n° ADELI**: -

n°RPPS: -

Mains publications:

November 16th, 2014:

Name: Martine DUCLOS

Functions: Professor, head of Sports Medicine department, CHU Clermont-Ferrand

Titles: MD, PhD

Establishment:
- Sports Medicine, CHU Clermont-Ferrand, Hôpital Gabriel Montpied, 58 rue Montalembert, 63000 Clermont-Ferrand

Research lab:

INSERM ☐ CNRS ☐ Others x

Address:
Human Nutrition Unit (UNH), Research Center In Human Nutrition (CRNH), Clermont-Ferrand, France

Phone: *+33 4* 73 75 16 64 ***Fax***: *+33 4* 73 75 16 61

e-mail: mduclos@chu-clermontferrand.fr

n° d'inscription au conseil de l'ordre: 63 5449 **n° ADELI**: 63 10 5449 1

n°RPPS:

Mains publications:

Duclos M, Oppert JM, Verges B, Coliche V, Gautier JF, Guezennec Y, Reach G, Strauch G; SFD diabetes and physical activity working group. Physical activity and type 2 diabetes. Recommandations of the SFD (Francophone Diabetes Society) diabetes and physical activity working group. Diabetes Metab. 2013;39(3):205-16. doi: 10.1016/j.diabet.2013.03.005. 5-year Impact factor 2013: **3.1**

Duclos M, Ouerdani A, Mormède P, Konsman JP. Food restriction-induced hyperactivity: addiction or adaptation to famine? Psychoneuroendocrinology. 2013;38(6):884-97. doi: 10.1016/j.psyneuen.2012.09.012. 5-year Impact factor 2013: **6.1**

Dutheil F, Lac G, Lesourd B, Chapier R, Walther G, Vinet A, Sapin V, Verney J, Ouchchane L, Duclos M, Obert P, Courteix D. Different modalities of exercise to reduce visceral fat mass and cardiovascular risk in metabolic syndrome: the RESOLVE* randomized trial. *International Journal of Cardiology*. 2013. doi:10.1016/j.ijcard.2013.05.012. Impact factor 2013: **7.1**

Dutheil F, Boudet G, Perrier C, Lac G, Ouchchane L, Chamoux A, Duclos M, Schmidt J. JOBSTRESS study: Comparison of heart rate variability in emergency physicians working a 24-hour shift or a 14-hour night shift - A randomized trial. *International Journal of Cardiology.* 2012 Jul;158(2):322-325. Impact factor 2013: **7.1**

Dutheil F, Trousselard M, Perrier C, Lac G, Chamoux A, Duclos M, Naughton G, Mnatzaganian G, Schmidt J. Urinary Interleukin-8 Is a Biomarker of Stress in Emergency Physicians, Especially with Advancing Age - The JOBSTRESS* Randomized Trial. *PLoS One*. 2013 Aug 19;8(8):e71658. doi: 10.1371/journal.pone.0071658. Impact factor 2013: **4.09**

November 16th, 2014:

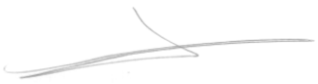

Name: Jean-Claude CHATARD

Functions: Professor of Exercise Physiology

Titles: MD, PhD

Establishment:
- Sports Medicine, Hôpital Bellevue, CHU Saint-Etienne, France

Research lab:

INSERM ☐ CNRS ☐ Others x

Address:
- Sports Medicine, Hôpital Bellevue, CHU Saint-Etienne, France

Phone*: +33* 6 64 51 60 17 ***Fax***:

e-mail: j.claude.chatard@chu-st-etienne.fr

n° d'inscription au conseil de l'ordre: 42-203 **n° ADELI**: 420 784 878

n°RPPS:

Mains publications:

Wilson MG, Chatard JC, Carre F, Hamilton B, Whyte GP, Sharma S, Chalabi H.Prevalence of electrocardiographic abnormalities in West-Asian and African male athletes. *Br J Sports Med*. 2012 Apr;46(5):341-7. doi: 10.1136/bjsm.2010.082743. 5-year Impact factor 2013: **4.34**

Wilson MG, Chatard JC, Hamilton B, Prasad SK, Carré F, Whyte GP, Chalabi H. Significance of deep T-wave inversions in an asymptomatic athlete with a family history of sudden death. *Clin J Sport Med*. 2011 Mar;21(2):138-40. doi: 10.1097/JSM.0b013e3182042a5b. Impact factor 2013: **1.60**

Schnitzler C, Heck G, Chatard JC, Ernwein V. A simple field test to assess endurance in inexperienced runners. *J Strength Cond Res*. 2010 Aug;24(8):2026-31. doi: 10.1519/JSC.0b013e3181d2c48d. Impact factor 2013: **1.86**

Bouassida A, Chatard JC, Chamari K, Zaouali M, Feki Y, Gharbi N, Zbidi A, Tabka Z. Effect of energy expenditure and training status on leptin response to sub-maximal cycling. *J Sports Sci Med*. 2009 Jun 1;8(2):190-6. 5-year Impact factor 2013: **3.49**

Portier H, Chatard JC, Filaire E, Jaunet-Devienne MF, Robert A, Guezennec CY. Effects of branched-chain amino acids supplementation on physiological and psychological performance during an offshore sailing race. *Eur J Appl Physiol*. 2008 Nov;104(5):787-94. doi: 10.1007/s00421-008-0832-5. 5-year Impact factor 2013: **2.30**

November 16th, 2014:

Name: Geraldine NAUGHTON

Functions: Professor

Titles: PhD

Establishment:
- School of Exercise Science (Pr G Naughton), Australian Catholic University, Locked Bag 4115 Fitzroy MDC VIC 3065, Melbourne, Australia

Research lab:

INSERM ☐ CNRS ☐ Others x

Address:
- School of Exercise Science (Pr G Naughton), Australian Catholic University, Locked Bag 4115 Fitzroy MDC VIC 3065, Melbourne, Australia

Phone: +61 3 9953 3034 ***Fax***: +61 3 9953 3095

e-mail: Geraldine.Naughton@acu.edu.au

n° d'inscription au conseil de l'ordre: - **n° ADELI**: -

n°RPPS: -

Mains publications:

Ollier M, Garcier JM, Naughton G, Chamoux A, Pereira B, Dutheil F. CT scan procedure for lung cancer screening in asbestos-exposed workers. Chest. 2014 Aug.5-year impact factor 2013: **6.42**

Ollier M, Chamoux A, Naughton G, Pereira B, Dutheil F. Chest computed tomography screening for lung cancer in asbestos occupational exposure: a systematic review and meta-analysis. *Chest*. 2014 Jan 30. doi: 10.1378/chest.13-2181. 5-year impact factor 2013: **6.42**

Dutheil F, Trousselard M, Perrier C, Lac G, Chamoux A, Duclos M, Naughton G, Mnatzaganian G, Schmidt J. Urinary Interleukin-8 Is a Biomarker of Stress in Emergency Physicians, Especially with Advancing Age - The JOBSTRESS* Randomized Trial. *PLoS One*. 2013 Aug 19;8(8):e71658. doi: 10.1371/journal.pone.0071658. Impact factor 2013: **4.09**

Crendal E, Dutheil F, Naughton G, McDonald T, Obert P. Increased myocardial dysfunction, dyssynchrony, and epicardial fat across the lifespan in healthy males. BMC Cardiovasc Disord. 2014 Aug 3;14(1):95. Impact factor 2014: **1.50**

Dutheil F, Walther G, Chapier R, Mnatzaganian G, Lesourd B, Naughton G, Verney J, Fogli A, Sapin V, Duclos M, Vinet A, Obert P, Courteix D, Lac G. Atherogenic subfractions of lipoproteins in the treatment of metabolic syndrome by physical activity and diet - the RESOLVE Trial. Lipids Health Dis. 2014 Jul 11;13(1):112. Impact factor 2014: **2.35**

November 16th, 2014:

Geraldine Naughton

Name: Bruno PEREIRA

Functions: biostatistician

Titles: PhD

Establishment:
- The Clinical Research and Innovation Direction, CHU Clermont-Ferrand, France

Research lab:

INSERM ☐ CNRS ☐ Others ☐

Adress:

Phone: *04 73 75 17 32* ***Fax***: *04 73 75 17 35*

e-mail: bpereira@chu-clermontferrand.fr

n° d'inscription au conseil de l'ordre: **n° ADELI**:

n°RPPS:

Mains publications:

Ollier M, Garcier JM, Naughton G, Chamoux A, Pereira B, Dutheil F. CT scan procedure for lung cancer screening in asbestos-exposed workers. Chest. 2014 Aug.5-year impact factor 2013: **6.42**

Ollier M, Chamoux A, Naughton G, Pereira B, Dutheil F. Chest computed tomography screening for lung cancer in asbestos occupational exposure: a systematic review and meta-analysis. *Chest*. 2014 Jan 30. doi: 10.1378/chest.13-2181. 5-year impact factor 2013: **6.42**

Tekath M, Dutheil F, Bellini R, Roche A, Pereira B, Naughton G, Chamoux A, Michel JL. Comparison of the ultra-low-dose Veo algorithm with the gold standard filtered back projection for detecting pulmonary asbestos-related conditions: a clinical observational study. *BMJ Open* 2014;4:e004980. doi:10.1136/bmjopen-2014-004980. Impact factor 2013: **1.8**

Mathieu S, Pereira B, Dubost JJ, Lusson JR, Soubrier M. No significant change in arterial stiffness in RA after 6 months and 1 year of rituximab treatment. *Rheumatology* (Oxford). 2012 Jun;51(6):1107-11.

Payet S, Pereira B, Soubrier M, Mathieu S. The effects of TNF-alpha blocking therapy on lipid levels in rheumatoid arthritis: a meta-analysis. *Semin Arthritis Rheum*. 2012 Feb;41(4):e4-5;

November 16th, 2014:

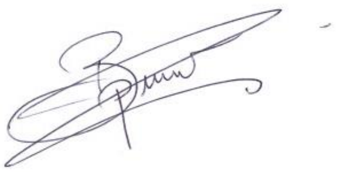

Supplement: Multimedia Appendix 1 [file resprot_v6i4e58_app1.pdf]
